# Implementation fidelity and acceptability of an intervention to improve vaccination uptake and child health in rural India: a mixed methods evaluation of a pilot cluster randomized controlled trial

**DOI:** 10.1186/s43058-020-00077-7

**Published:** 2020-10-08

**Authors:** Myriam Cielo Pérez, Dinesh Chandra, Georges Koné, Rohit Singh, Valery Ridde, Marie-Pierre Sylvestre, Aaditeshwar Seth, Mira Johri

**Affiliations:** 1 Centre de Recherche du Centre Hospitalier de l’Université de Montréal (CRCHUM), Tour Saint-Antoine, Porte S03-102, 850, rue St-Denis, Montréal, Québec H2X 0A9 Canada; 2 Département de Médicine Sociale et Préventive, École de Santé Publique (ESPUM), Université de Montréal, Montréal, Québec Canada; 3 Independent Consultant Tika Vaani, New Delhi, India; 4 Management Sciences for Health (MSH)/USAID, Port-au-Prince, Haiti; 5 Gram Vaani Community Media Pvt. Ltd., New Delhi, India; 6 Centre de recherche en santé publique, Université de Montréal, 7101 avenue du Parc, Montréal, Québec Canada; 7 IRD (French Institute for Research on Sustainable Development), CEPED (IRD-Université Paris), Université de Paris, ERL INSERM SAGESUD, 45 rue des Saints-Pères, 75006 Paris, France; 8 Department of Computer Science, Indian Institute of Technology Delhi, New Delhi, India; 9 Département de gestion, d’évaluation, et de politique de santé, École de Santé Publique (ESPUM), Université de Montréal, Montréal, Québec Canada

**Keywords:** Pilot study, Child health, mHealth program, Implementation Science, Implementation fidelity, Adherence, Process evaluation, mixed methods evaluation, Developing countries, Global health

## Abstract

**Background:**

The Tika Vaani intervention, an initiative to improve basic health knowledge and empower beneficiaries to improve vaccination uptake and child health for underserved rural populations in India, was assessed in a pilot cluster randomized trial. The intervention was delivered through two strategies: mHealth (using mobile phones to send vaccination reminders and audio-based messages) and community mobilization (face-to-face meetings) in rural Indian villages from January to September 2018. We assessed acceptability and implementation fidelity to determine whether the intervention delivered in the pilot trial can be implemented at a larger scale.

**Methods:**

We adapted the Conceptual Framework for implementation fidelity to assess acceptability and fidelity of the pilot interventions using a mixed methods design. Quantitative data sources include a structured checklist, household surveys, and mobile phone call patterns. Qualitative data came from field observations, intervention records, semi-structured interviews and focus groups with project recipients and implementers. Quantitative analyses assessed whether activities were implemented as planned, using descriptive statistics to describe participant characteristics and the percentage distribution of activities. Qualitative data were analyzed using content analysis and in the light of the implementation fidelity model to explore moderating factors and to determine how well the intervention was received.

**Results:**

Findings demonstrated high (86.7%) implementation fidelity. A total of 94% of the target population benefited from the intervention by participating in a face-to-face group meeting or via mobile phone. The participants felt that the strategies were useful means for obtaining information. The clarity of the intervention theory, the motivation, and commitment of the implementers as well as the periodic meetings of the supervisors largely explain the high level of fidelity obtained. Geographic distance, access to a mobile phone, level of education, and gender norms are contextual factors that contributed to heterogeneity in participation.

**Conclusions:**

Although the intervention was evaluated in the context of a randomized trial that could explain the high level of fidelity obtained, this evaluation provides confirmatory evidence that the results of the study reflect the underlying theory. The mobile platform coupled with community mobilization was well-received by the participants and could be a useful way to improve health knowledge and change behavior.

**Trial registration:**

ISRCTN 44840759 (22 April 2018)

Contribution to the literature

- Fidelity assessment is a useful method for pilot trials to strengthen community interventions, improve their sustainability, and replicate them in other contexts.
- The implementation of community interventions may be affected by diverse factors, including cultural and socioeconomic dimensions, gender, and education. These factors should be systematically studied because they can affect program adherence.
- The training, understanding, and credibility of implementers and the availability of resources and feedback from supervisors are key understudied factors contributing to successful implementation and high implementation fidelity.

## Background

Public health interventions should be based on the best available evidence, and randomized field experiments are considered the strongest evaluation design by many. However, these interventions may vary during implementation due to diverse factors related to the complexity of the intervention, context, participants, and implementers. Their evaluation is, therefore, usually complex and challenging [1]. These factors need to be considered at the time of evaluation and before replication in other contexts [2–5]. A pilot study is a smaller-sized study that aims to investigate the feasibility of the crucial components of the main study and guide the planning of a large-scale intervention [6]. Implementation science is a field of methods to promote the systematic uptake of research findings into routine practice [7]. Pilot studies play an essential role in the development of cluster randomized trials (CRT). They can also contribute to the implementation of science goals by helping to identify the factors that may affect intervention effectiveness before conducting a large-scale study. Applying an implementation science lens to pilot studies is crucial to identify the factors that restrict or facilitate their replication [8–10] and is particularly necessary in low-income countries, which need to optimize resources to benefit as many people as possible and improve public health.

Implementation fidelity, sometimes called adherence or integrity, refers to the degree to which an intervention is delivered as planned [11]. Implementation fidelity is crucial for the successful application of evidence-based interventions [12–14]. Fidelity assessment permits the following: (i) the assessment of cause and effect relationships in studies of complex interventions; (ii) the explanation of variations in study results; (iii) the identification of components that require improvement; (iv) the identification of potential barriers to the successful delivery of interventions at scale; and (v) the collection of information needed to identify problems, propose solutions, and help increase the chances of success of these interventions [11–17].

### Context of the Tika Vaani intervention

It is estimated that among the 19.5 million children worldwide who did not receive all basic vaccines in the first year of life in 2016, 16% were from India [18]**.** India has achieved considerable improvements in child vaccination coverage; however, significant challenges to closing the immunization gap remain, especially those related to beneficiary demand for immunization [19]. Sociodemographic factors, levels of knowledge, and public beliefs may affect vaccination coverage in children [18, 19]. Educational approaches targeting the general population and adapted to local beliefs are necessary to address these barriers [19]. Vaccination coverage remains especially low in Uttar Pradesh [18].

The Tika Vaani (“voice of vaccination” in Hindi) intervention was designed to address this issue by using an interactive mobile platform combined with community mobilization to reduce the gap in childhood vaccination. This intervention primarily aimed to increase immunization coverage among children 0 to 2 years old living in rural areas of Uttar Pradesh, India. In addition, to strengthen primary health care, the intervention seeks to improve health literacy among community members, health workers, and families with young children.

Mobile health (mHealth) is defined as medical and public health practice supported by mobile devices [20]. Despite the inherent challenges, especially when combined with non-mHealth interventions, mHealth is an effective tool to improve maternal and child health in low- and middle-income countries (LMICs) [21]. In particular, interactive voice response (IVR) technology has become an increasingly popular approach for sending educational messages about behavior change and has been widely used in rural contexts in India to help illiterate communities improve maternal and child health [22, 23]. A recent study pointed out that although literacy in India has improved over the past three decades, Uttar Pradesh is one of the states with the highest rates of illiteracy [24], and women are especially disadvantaged. The 23% literacy gender gap in the state is more than four times the 2016 global average. According to the most recent round of the National Family Health Survey (NFHS, 2015), in Uttar Pradesh, 61% of women are literate in comparison to 82.4% of men [25]. In Uttar Pradesh, 55% of rural households and 78% of urban households own a mobile phone [26]. The rapid growth in mobile phone coverage in India is an excellent opportunity to launch mobile-learning programs that could reduce health disparities and improve healthcare outcomes. In addition, social mobilization is recognized as a key health-promotion strategy used by LMICs to promote vaccination [27]. The Tika Vaani intervention uses both the strategies to overcome illiteracy barriers, reach users who do not have a mobile phone, and motivate telephone users to participate in achieving their goals.

### Fidelity of the Tika Vaani intervention

The Tika Vaani intervention was evaluated through a pilot CRT study conducted in rural villages of Hardoi district, Uttar Pradesh, between January and September 2018. A quantitative assessment of study feasibility, intervention uptake, coverage, and early impacts is provided in the main pilot trial report [28]. Overall, quantitative results showed that all predetermined standards for feasibility were met. Intervention uptake was extremely high, and basic health knowledge was significantly higher among the intervention group. This mixed methods study complements the main analysis by addressing implementation research questions that help to shed light on whether the program requires changes before further assessment and large-scale replication. The specific objectives were to (i) measure the level of implementation fidelity of the interventions (quantitative approach), (ii) assess factors that could potentially influence fidelity (qualitative approach), and (iii) determine whether the proposed interventions are acceptable to participants (qualitative and quantitative approach, see Table 1).

**Table 1:** Outcome variables and data sources for the Tika Vaani pilot study

Outcome	Definition	Approach	Analysis population	Data sources
Primary				
Feasibility of the future main study	*Ex-ante criteria related to key study processes and measures*	*Quantitative*	*Intervention and control groups*	*Reported in main study*
Uptake of interventions (adoption)	*Evidence of participation in the new interventions*	*Quantitative*	*Intervention group*	*Reported in main study*
Secondary				
Acceptability*	Perception among stakeholders that an intervention is agreeable, suitable, relevant, useful, and credible.	Mixed methods	Intervention group	QUANT IVR platform (mHealth); Household surveys QUAL Semi-structured interviews Discussion groups
Fidelity*	Ability to deliver the interventions as planned	Mixed methods	Intervention group (some information from controls)	QUANT Project records Structured observation with checklist; IVR platform (mHealth); Household surveys QUAL Semi-structured interviews Discussion groups Field observation Document review
Coverage	*The degree to which a (sub)population that is eligible to benefit from an intervention actually receives it*	*Quantitative*	*Intervention group*	*Reported in main study*
Program theory	*Anticipated changes in knowledge, attitudes, and practices of end users*	*Quantitative*	*Intervention and control groups*	*Reported in main study*

Adapted from Peters et al., 2013

*IVR* interactive voice response, *mHealth* mobile health, *QUANT* quantitative, *QUAL* qualitative

*Outcomes analyzed for the present study

## Methods

### The Tika Vaani intervention

The study interventions took place over a 3-month period and offered social and behavior change communication (SBCC) for members of the general public in rural Indian villages addressing topics related to child health. The intervention is described in detail in the main article [28]. The program rationale and design were iteratively developed through a comprehensive review of the literature, formative evaluations [29], and training of implementers to develop activities that responded to local needs and were sustainable in the long term [30]. SBCC materials were delivered through two channels: (1) community (face-to-face) mobilization strategy, including one large introductory meeting offered to each village and three small group meetings offered to each participant; and (2) individual audio messages via mobile phone (mHealth) strategy delivered through the IVR system. For the mHealth component, automated dial-outs featuring entertaining educational audio capsules and voice immunization reminders were favored, and “on-demand” access to all content was offered. Table 2 summarizes the activities, objectives, and beneficiaries of each key component of the intervention. To facilitate implementation, monitoring, and evaluation, a well-defined role was established for each of the actors involved in the intervention (Table 3) [31]. More details about the interventions and delivery processes are reported in the associated TiDIER Checklist [28]. All intervention components were offered free of charge to end users. The control group received all standard health services provided by the Government of India, including monthly Village Health and Nutrition Days (VHND).

**Table 2:** Intervention components and beneficiaries for the Tika Vaani intervention

Key components	Activities per village	Purpose	General public	Primary caregivers and families of children 0 to 12 months of age	Frontline workers*
**Community mobilization (face-to-face strategy)**	-Large introductory meeting (*n* = 1)	To inform the community about the intervention and invite participation	x	x	x
	-Small group meetings (*n* = 3)	To educate and reinforce basic health knowledge	x	x	x
**Messages via mobile phone (mHealth strategy)**	‟Pushed” edutainment and summary capsules via mobile phone (*n* = 13)	To educate and reinforce basic health knowledge.	x	x	x
	Vaccination “reminders” via mobile phone	To inform when the child's vaccination is due		x	
	‟On-demand” (callback) access via mobile phone to content through the IVR portal	To provide convenient access	x	x	x

*Considered for the present study, accredited social health activist [ASHA], *Anganwadi* workers [AWWs], and AWW helpers (Sahaika). All interventions were offered free-of-cost to end users

**Table 3:** Role of the implementers and the research group in the Tika Vaani intervention

Actors		Role
Implementers	Field staff	Conduct a series of 4 community meetings: - A large group introductory meeting to inform the purpose the intervention and encouraging participation; to promote the Tika Vaani intervention by painting the logo, and telephone number on village walls and distribute pamphlets (sheets) and stickers containing the Tika Vaani phone number. -Three small group meetings held at monthly intervals to discuss the themes assigned for each meeting in order to assess the interest, acceptability, and understandability of the information capsules and comprehension and retention of key messages.
Research group	Field staff coordinator Intervention coordinator	- Monitor the progress of activities carried out in the field - Provide technical support.
	-Interactive voice -response (IVR) system coordinator	- Monitor the progress of information capsules delivered - Provide technical support.

### Study framework to assess implementation fidelity

We used the conceptual framework proposed by Carroll and colleagues [11] as modified by Hasson [16] to evaluate the fidelity of the Tika Vaani intervention. This framework, useful for assessing the fidelity of complex interventions [5], allows us to assess whether (i) the activities were implemented as planned (content), (ii) the number of planned activities and the designated area were respected (coverage), and (iii) the activities were delivered with the regularity planned by its designers and occurred over a specified period (frequency and duration). This framework also allows us to explore whether there are specific moderating factors that can explain the degree of fidelity obtained. The framework used does not consider evaluating the control group, but its evaluation is crucial to determine the degree of treatment differentiation, which is the systematic variance that is expected to account for any differences in outcomes [32, 33]. We investigated whether members of the control group participated to evaluate possible contamination between study groups. This fidelity assessment used a Mixed Methods Concurrent Triangulation design [34] because the research question focused on a single phenomenon—the fidelity of the Tika Vaani intervention—and measured fidelity and factors that could explain the achieved level of fidelity, which could not be adequately assessed by either qualitative or quantitative methods alone. Based on the GRAMMS checklist, a guide to improve Good Reporting of A Mixed Methods Study [35], we provide information about the fidelity assessment of the Tika Vaani intervention through a Mixed-Methods Concurrent Triangulation design (Additional file 1).

### Data collection

Qualitative and quantitative data were obtained from multiple sources and methods (Table 4). Data were collected between April and August 2018. The qualitative tools were translated to Hindi (the local language) and validated before use by field team members who were native Hindi speakers residing in the study area. To preserve meaning in the translation process, two bilingual authors (DC, MJ) fluent in Hindi and English with a detailed understanding of the study context, including the cultural characteristics of the participants, led this process. Qualitative data were collected by a research assistant not involved in the intervention. The triangulation of these methods was used to strengthen the validity of the constructs, ensure consistency, confirm data reliability, and increase the validity of the study [34, 36].

**Table 4:** Overview the conceptual framework to assess acceptability, fidelity implementation, and type of data collection for each dimension

Definition	Purpose of the information	Type of data
Fidelity components		
Content Defined as an attempt to establish the “active ingredients” of the intervention, for example, in a theory of change or logic model, and assess whether they have been delivered as planned	Number of components implemented as planned Community mobilization strategy # community meetings mHealth strategy #“pushed” edutainment capsules #“pushed” vaccination reminder messages	Quantitative ^a^
Coverage Refers to the degree to which all persons who met study inclusion criteria received the intervention	Community mobilization strategy # and characteristics of individuals (general population and target group) attending the different scheduled sessions mHealth strategy # and characteristics of individuals using the IVR platform by content type (edutainment (general population and target group), vaccination reminders (target group only))	
Frequency Refers to whether the intervention was delivered with the regularity or frequency planned by its designers.	Number of activities delivered defined in time and frequency according to the scheduled calendar: Community mobilization strategy # community meetings mHealth strategy # “pushed” edutainment capsules # “pushed” vaccination reminder messages ‟On-demand” to content through the IVR portal	
Duration Establishes whether the intervention was delivered with the duration planned by its designers		
Moderating factors		
Comprehensiveness of intervention description Factors such as the degree of intervention complexity, and whether the intervention description is complete or incomplete, vague or clear, may influence the degree of implementation fidelity	To evaluate the implementers' understanding of: -the theory of intervention -the activities and resources allocated to the different components of the intervention -the role in the intervention *This dimension is evaluated in three moments: (i) before beginning the data collection from intervention records, (ii) at the end of the implementation based on discussion groups and interviews with the intervention implementers and (iii) through feedback meetings with program designers*	Qualitative ^b^
Strategies to facilitate implementation Several support strategies may be used to optimize and to standardize implementation fidelity	According to the perspective of the implementers: What were the strategies that facilitated the implementation? What were the facilitating elements and the challenges encountered during the implementation phase?	
Quality of delivery Concerns whether an intervention is delivered in a way that increases the likelihood of achieving the desires health outcomes	According to the perspective of the implementers: To assess the quality of the material used, the delivery of the content of the intervention and the participation of the participant	
Participant responsiveness Intervention uptake depends on its acceptance by and acceptability to those receiving it. Low participant involvement or responsiveness may negatively impact intervention fidelity	To know the acceptability and usefulness of the activities and the different key messages delivered and according to the perspective the -Frontline workers -General public and primary caregivers and families of children 0 to 12 months of age To understand the reasons for non-participation of member of the target group in the proposed activities: -Community mobilization (face-to-face) activities -Messages via mobile phone (mHealth) activities	
Recruitment* Refers to procedures that were used to attract potential program participants.	According to the perspective of the implementers: To assess recruitment process, recruitment strategies and challenges to attract participants in each group (to compare the level of fidelity achieved in each village).	
Context* Refers to surrounding social systems, such as structures and cultures of organizations and groups, and historical and concurrent activities and events.	-Reasons for any deviation from the planned activities according to the point of view of the implementers -To assess which contextual factors influence the fidelity obtained in the different components of the intervention -Information on context factors regarding the delivery and receipt of the intervention in the different villages -The actions of the Government of India to provide primary health care in rural areas	
Control group Monitoring of events in the control group	-Components of the intervention that took place in the control group during the intervention period. -Strategies adopted to prevent contamination in the control group.	

Adapted from Carrol et al. [11] and these components* added by Hasson [16]

^a^ Research methods: structured observation with checklist, survey records and data from the interactive voice response (IVR) system and household surveys. Data source: implementers, administrative records of the intervention Tika Vaani, and the mobile platform IVR

^b^ Research methods: semi-structured interviews, discussion groups, documentary review and field observation. Data source implementers, frontline workers, general public and primary caregivers and families of children 0 to 12 months of age, records of the intervention Tika Vaani, and the mobile platform IVR, and journal of the main author

#### Quantitative data

Structured observation with a checklist was used by each implementer to verify if planned activities were implemented as specified in terms of content, coverage, duration, and frequency. For each planned activity, we evaluated whether it was Implemented as planned (I), Modified (M), or Cancelled (C). Further, implementers were asked to note if any activity was added during implementation (Additional file 2). Data from the IVR system and household surveys conducted at the study baseline and end line in both the study groups were also consulted to understand the degree of fidelity obtained.

#### Qualitative data

Qualitative data were obtained from semi-structured interviews, focus group discussions, semi-structured observations (Additional file 3), and analysis of records to provide complementary information on different factors that affected the fidelity of implementation and adjustments to the program.

#### Participants

All implementers of the intervention (*n* = 8) provided data from semi-structured interviews and discussion groups to identify possible modifications, difficulties encountered during the implementation phase, and strategies to address these difficulties and understand the program rationale. The semi-structured interviews (*n* = 8) and two discussion groups with implementers lasted 30–60 min and were recorded and conducted in a confidential setting.

#### Community

Small discussion groups

We analyzed information collected from 915 participants during 96 small discussion groups (Additional file 4) on the program rationale, mobile platform, records, and feedback that the lead author obtained during the development and implementation phases. Seven meetings were conducted with the group coordinator to clarify and validate information.

#### Community exit meetings

Community discussions (*n* = 25) were held after the end line survey in all intervention villages to provide an opportunity for partner communities to express their views. A purposive sample based on the characteristics of the target group for the intervention (parents of children younger than 2 years), regardless of participation, and who were available to attend a discussion group, was used to recruit participants for the qualitative phase [37, 38]. However, all members of the community were invited to give their feedback on the intervention. Several meetings were held in each village to ensure convenience and facilitate discussion. We used a structured interview guide to conduct meetings and a pre-defined data collection tool including closed-form responses and some space for open comments to record feedback. The discussion groups aimed to understand the participants’ perceptions, understanding, and acceptance of the activities. Discussions were held in a place easily accessible to participants and lasted approximately 60 to 90 min. In total, in the 13 intervention villages, 25 exit meetings (three meetings were attended by men and 22 by women) were held with an average of 20 participants (*n* = 292 participants) per meeting.

#### Frontline workers

A purposive sample comprising personnel from the target area who were available to attend interviews was recruited [37, 38]. Semi-structured interviews with frontline workers (*n* = 17) lasting 30–40 min were performed at the workplace to examine these professionals’ perceptions of the program.

### Data analysis

#### Quantitative data analysis

A matrix was created to assess whether activities were modified, canceled, or implemented as planned. For each component, the number of implemented activities relative to that of planned activities was calculated and multiplied by 100 to obtain the percentage representing the degree of fidelity. The percentages were summed up to obtain the overall degree of fidelity of the intervention. Although no specific guideline was used to define the optimal degree, values between 80 and 100% were typically considered high [39, 40]. The following scoring categories were used in this study: 80–100%, high; 79–51%, moderate; and ≤ 50%, low.

Data from questionnaires, intervention records, and mobile platform records were analyzed by a researcher not involved in the project using STATA software version 15. Subsequently, data considered relevant to measuring fidelity was validated and analyzed by the first author (not involved in the implementation phase). Descriptive statistics were used to describe the characteristics of the participants and the distribution of activities.

#### Qualitative data analysis

Qualitative data from semi-structured interviews and discussion groups were transcribed and translated from Hindi to English by a researcher not involved in the interventions. Interviews were chosen at random to assess the veracity of the translation. The Framework Method was used for management and analysis of qualitative data [41, 42]. This phase included four stages: (i) transcription and translation of the edited data in word format, and reading of the transcribed material to familiarize the researchers with the data; (ii) data coding following two approaches: deductive, to identify the fidelity parameters of the conceptual framework, and inductive, to identify emerging information [43]; (iii) we kept the quotation that we considered the most relevant to assess implementation fidelity and assigned them codes according to our conceptual framework, using QDA Miner software to store and organize the data making it accessible for the analysis process; (iv) we use a matrix to summarize the data. The analysis included reading and evaluating the connections between codes by the first author to obtain a complete picture of the fidelity assessment until data saturation was reached. Subsequently, the information was verified and validated by a second author to resolve divergent interpretations and obtain an agreement on the identified findings.

### Data integration

**Fig. 1:**
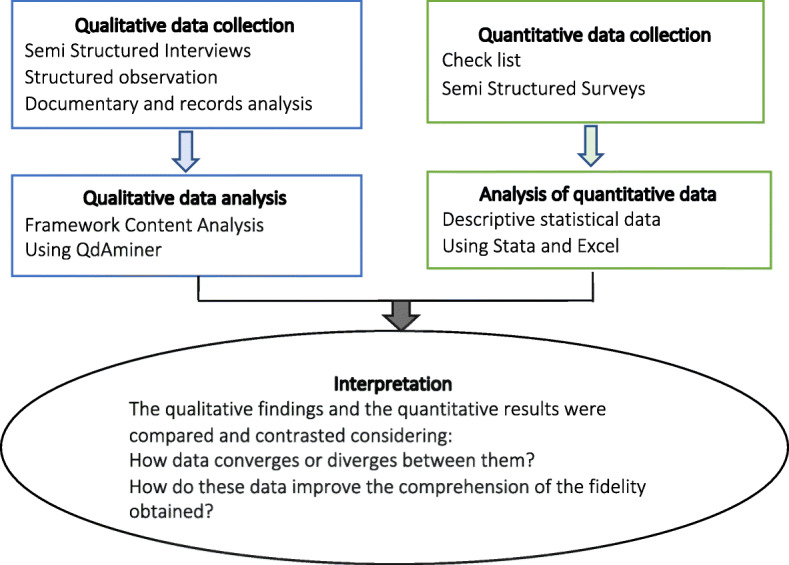
Mixed methods study flow diagram of collection, analysis, and integration of study data

Although most data sources were qualitative, both data types had the same priority in this study. Quantitative and qualitative data were collected, analyzed, and integrated in parallel (Mixed-Methods Concurrent Triangulation design) to shed light on the degree of fidelity obtained and increase the validity of our results and conclusions [44, 45] (Fig. 1).

## Results

The results are presented according to our conceptual framework. However, comprehensiveness of the policy description (a moderating factor) is presented first to give greater clarity about the intervention evaluated. Additional information relevant to understanding the level of fidelity achieved is provided (Additional file 5).

### Comprehensiveness of the policy description

**Fig. 2:**
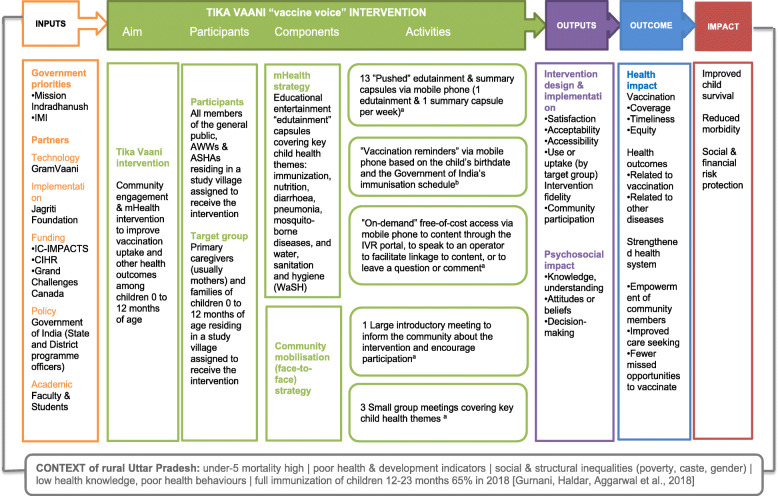
Logic model for the “Tika Vaani” to improve vaccination uptake and other health outcomes. a Activity directed towards target group households but open to all village residents, ASHAs, and AWWs. b Service offered exclusively to target group households

The policy description followed the Template for Intervention Description and Replication (TIDieR) guidelines [46]. During the study’s formative phase, implementers received information and training about the theory of the program, its goals, and the strategies for achieving these goals. In addition, they participated in the development of the project. Discussion groups (*n* = 2) and interviews (*n* = 8) with the implementers allow us to corroborate their understanding and their belief in the program objectives and intervention components, lending validity to the intervention logic model (Fig. 2).

## Adherence

### Content

According to different activities of the intervention, the results demonstrated that the fidelity of the intervention was high, with an overall rate of 86.7% (Table 5). For the face-to-face strategy, two of the 13 villages did not have the *large introductory meeting*. In one village, this omission was in accordance with the pre-established intervention protocol due to the small population of the village. In another village, the attempt to organize a meeting was unsuccessful.

**Table 5:** Content dimension

Key components	Activities for selected villages	Tasks of each planned activity	Planned activity	Implemented activity	% fidelity achieved
Community mobilization (face-to-face strategy) 90.3%	Large introductory meeting	(a) First contact with the community leader to get permission. (b) To visit each target household to invite them to the meeting (c) Collected mobile numbers from the target households (d) Collected mobile numbers of the people who wish to receive more health information through the platform. (e) Invite ASHA and AWW to participate in the big meeting (f) Paint a wall with the logo and the number TV (g) Pasted TV poster in the villages (h) TV team gave their introduction to community in the introductory meetings (i) Demonstration about how to access the TV platform (j) Distribution the stickers (sheets) with information on the TV phone number (k) Street play	13	11	84.6%
	Small group meetings	(a). To visit each target household to invite them to the small group meetings (b) To collect mobile numbers from the target households who wish to receive health information through the platform (This activity was done only during 1st and 2nd small group meetings) (c) To visit ASHA and AWW workers to invite them for small group meetings (d) Wall painting the logo and the number TV in the villages (e) To Paste TV poster in the villages (f) Our team also collected information about newborn children during 1st and 2nd small group meeting. Small group activities: (g) Introduction activity with the meeting participants (h) Use a guide sheet to know the experiences and the perception of the community regarding the TV Intervention and participants attending. (i) To play reminder capsule or straight content and then TV team discussed these capsules with the participants. (j) TV number demonstration (k) To distribute TV number slips	**GD #1** 13	**GD #1** 13	**GD #1** 100%
			**GD #2** 13	**GD #2** 11	**GD #2** 84.6%
			**GD #3** 13	**GD #3** 12	**GD #3** 92%
*Messages via mobile phone (mHealth strategy)* 83.15%	“Pushed” edutainment and summary capsules	- Transmit 13 educational capsules and 13 reminder messages during implementation period	26	26	100%
	Vaccination “reminders”	-Reminder messages for each target family to remember vaccination period	184	122	66.3%
	‟On-demand” free-of-cost access via mobile phone to content through the IVR portal	-The entire population was invited to participate spontaneously to dial the number to obtain information on the different capsules	**–**	**–**	**–**

One (1) village there were only 4 households and, in another (1) village, the team tried to organize the meeting, but people didn’t come to the meeting.—R-FDBK

A total of 96 *small group meetings* were held throughout the study period. The number of meetings per village depended on that of target families in the area. To collect additional data for research purposes, target families that lived far and did not attend the meeting were visited at their homes. For the mHealth strategy, *messages via mobile phone* were provided to participants with mobile phones who agreed to receive and send messages through the mobile platform.

### Coverage

This study was conducted in 13 villages and included 184 families, which composed the target group. All residents (*n* = 8516) from these villages and 33 frontline workers from the intervention group (12 ASHAs, 11 AWWs, 10 AWW helpers) were invited to participate. A total of 94% (173/184) of the target population benefited from the program either by participating in the face-to-face strategy (community mobilization activities) or by mHealth strategy (mobile phone messages or listening to at least 80% of the information) through different types of calls: “pushed” edutainment and summary capsules, vaccination reminder, or on-demand (callback). Table 6 presents the coverage by the different study participants.

**Table 6:** Coverage dimension

Key components	Activities	General public	Primary caregivers and families of children 0 to 12 months of age	Frontline workers
Community mobilization (face-to-face strategy)	Large introductory meeting	19% (1692/8516)	100% (184/184)	30% (10/33)
	Small group meetings	GD #1: 184 GD #2: 269 GD #3: 225	GD #1: 45.6% (84/184) GD #2: 46.7% (86/184) GD #3: 40.2% (74/184)	GD #1: 15% (5/33) GD #2: 36% (12/33) GD #3: 21% (7/33)
*Messages via mobile phone (mHealth strategy)*	“Pushed” edutainment and summary capsules	–	44.6% (82/184) of households listened to at least one OBD	85% (28/33)
	Vaccination “reminders”	–	66.3% (122/184) received at least 1VHD reminder message	–
	Use of the IVR system to access health information (on-demand access [callback])	–	29.3% (54/184) of households received at least 1 callback	88% (29/33) listened to at least one OBD

#### Community mobilization (face-to-face strategy)

Of the 1694 participants in the *large introductory meeting*, 75% (*n* = 1264) were women and children, and 25% (*n* = 430) were men. Although men were highly interested in the interventions, the rate of participation was low because most actions were conducted during the day, when they were working.

**Fig. 3:**
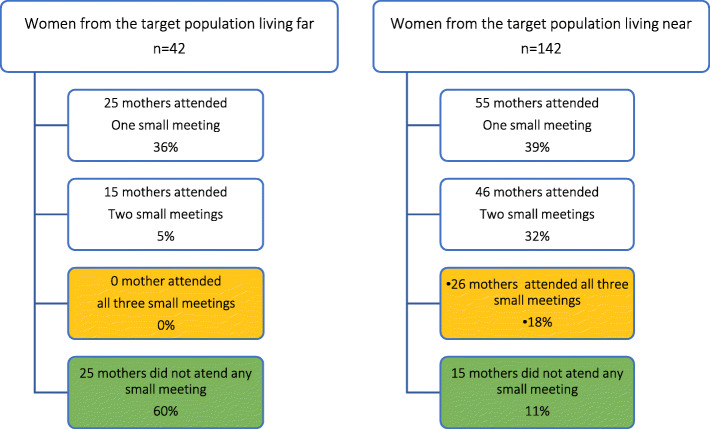
Participation of mothers to the small group meetings

**Fig. 4:**
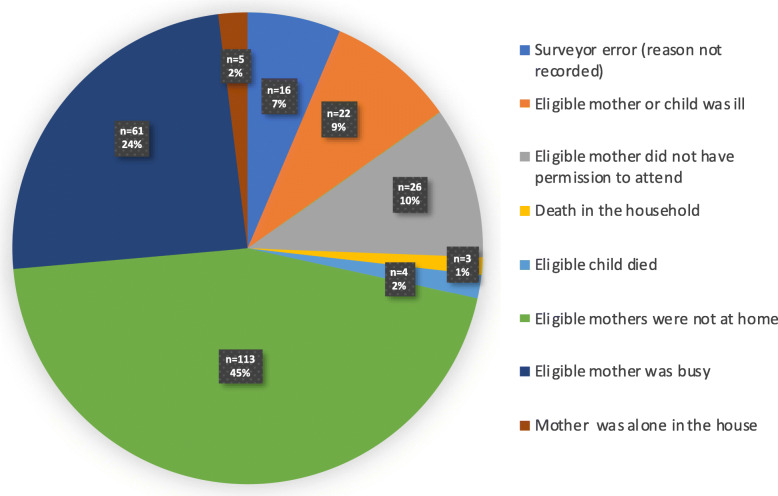
Reasons for non-participation of mothers in small group meetings

Throughout the study period, 428 people attended at least one *small group meeting*. Of them, 66% (284/428) came from the community, and 34% (144/428) were caregivers (representing 78.2% of the target group households (144/184)). The target population who lived far from the intervention site constituted 22.8% (42/184), and their participation was low (Fig. 3). The main reason for the non-participation of mothers was being away from home at the time of meetings (Fig. 4). Frontline workers did not attend all meetings because they were not present at the time of the visit to the community. The non-remuneration of this group to attend meetings might also explain the low rate of participation.

#### Messages via mobile phone (mHealth strategy)

**Fig. 5:**
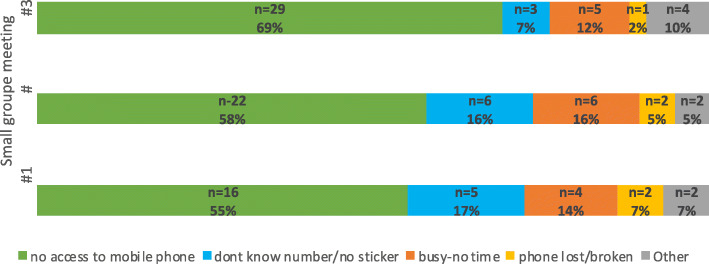
Reasons given by meeting participants for not listening to the educational capsules via mobile phone

The population attending small meetings benefited from “pushed” edutainment and summary capsules. Among the target population, 70.7% (130/184) received at least one type of “pushed” edutainment and summary capsules, vaccination reminder, or on-demand (callback). For ‟on-demand” (callback), the lack of access to a telephone was the main reason for not receiving calls (Fig. 5). Of the 29 frontline workers with a mobile phone, 100% (29/29) heard at least one capsule, and 34.5% (10/29) received at least one call through the platform.

### Frequency and duration

The program began a few weeks later than scheduled because of local factors: failure to deliver the information capsules on time, technical problems related to the mobile platform, and official religious holidays. However, there were no significant changes to the interventions during the study period.

## Moderating factors

### Strategies to facilitate implementation

During the study’s formative phase, a manual containing key concepts for working with the community—together with a schedule of intervention activities—was used to develop the necessary skills and standardize the knowledge of the field staff. The implementers of the intervention felt that training and training manuals facilitated implementation:

How we should fill formats and with whom to fill them. We also received training on how to demonstrate Tika Vaani to reach the people of the village…all this has simplified our work.—C22

What and how to do after reaching a village. The manual and guidelines provided were very useful.—C22

Field experience, motivation, periodic meetings, field notes, and the presence of frontline workers were factors that facilitated community contacts and interactions with participants.

### Quality of delivery

The information capsules were evaluated and tested to ensure the quality of the sound and content. During the implementation phase, community events were monitored through meetings with supervisors, and messages via mobile phone were evaluated by the technical team in charge of the mHealth component of the project (RS, AS).

### Participant responsiveness

The participants felt that the strategies used were useful means for obtaining information because it was easy to access from home, available on demand, and both easy to understand and entertaining. In addition, they recognize that the information obtained is important for their health.

Yes, method is good [getting information through phones and community meetings]. You people come, do meetings and provide information…We get information while sitting at home.—Community#12

An eligible mother didn’t use to take her children for vaccination but after listing stories on TikaVaani platform now she takes her children for vaccination.—Community18

The content of the calls was considered useful, entertaining, and easily understood by the participants. Moreover, they considered reminder messages about childhood vaccination useful.

There is no other method [reminding families on child vaccination through calls], phone is the best method.—Community#2

This information [reminding families on child vaccination through calls] is very helpful for all.—Community#12

From meetings and mHealth capsules, the participants received information on how to recognize the main symptoms of conditions such as diarrhea, pneumonia, dengue, and chikungunya, and manage these diseases.

**Fig. 6:**
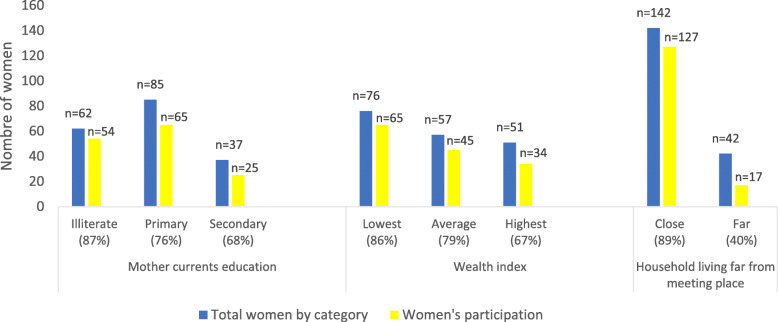
Factors influencing participation of women in community mobilization activities *n* = 184

**Fig. 7:**
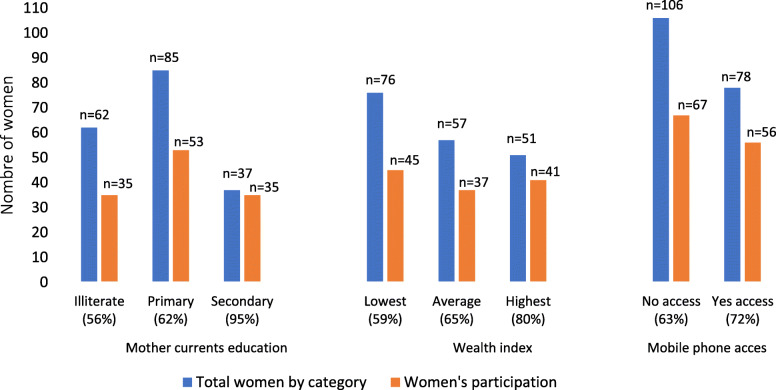
Factors influencing participation of women in educational capsules via mobile phone *n* = 184

The participation of mothers in different activities was influenced by multiple factors (Figs. 3, 4, and 5). The results showed that the benefits of the program were affected by the level of education and socioeconomic status, whereas sociodemographic characteristics and access to mobile phones directly affected access to information through meetings and mobile devices, respectively. Among the women who attended small group meetings, 29% (54/184) were illiterate, 35% (65/184) were from the poorest quintile, and 69% (127/184) lived close to the meeting place (Fig. 6). Among the women who accessed information through mobile devices, 19% (35/184) were illiterate, 24% (45/184) were from the poorest quintile, and 30% (56/184) had direct mobile phone access (Fig. 7). Among the women who lacked access to a mobile phone, 63% (67/106) were able to access the information through a family member’s mobile phone either to listen to vaccination reminders and edutainment capsules or to communicate with the platform at some point in the intervention period.

Frontline workers reported that mothers changed their behavior and were more aware of childhood vaccinations and adherence to the vaccination schedule.

Complete vaccination give full protection from diseases, Timely vaccination is important to protect from diseases, there are side effects of vaccines but not serious.—Community15

Women say that their children did not get all the vaccine because vaccines are not written in the card. People are becoming aware... After listening content over this number, people started coming for vaccination.—HW8

Now vaccination is happening successful people take their children immediately if their child is sick… Now everyone is coming for vaccination.—HW3

In view of the benefits to the community, frontline workers reported that the intervention should continue over time and were interested in receiving further training.

Your team should keep coming to make difference among people.—HW8

Our village people liked the TikaVaani program. Team should be sent to every place to spread information.—HW2

### Recruitment

The recruitment process was the same for the 13 villages; nonetheless, certain factors such as the level of education, time constraints, or interest of the participants limited this process.

Mobilizing men and women for meetings is the most challenging task.—C66

People did not have time to attend meetings. It was difficult to mobilize people to attend meetings.—C44

[It was not easy] “Because some villagers were less educated, it was not easy.”—C66

Some of the villagers are educated people who motivate others to attend meetings and teach them how to use the Tika Vaani number. Due to which our work becomes much easier.—C33

However, the commitment and motivation of the implementers to carry out the different activities led them to develop strategies to attract participants and keep their attention during the planned activities:

We tried to organize meetings in a quiet place. If during the meeting some children created a disturbance, then one of the Tika Vaani team members took the children to a different location and did some activities with them.—C33

To keep the attention of participants, we used speakers and storytelling methods to disseminate information about Tiki Vaani.—C77

Despite the obstacles encountered to attract participants, implementers and frontline workers found that the intervention was gaining credibility among the participants.

There was a substantial change in people’s demeanor. Those people who did not trust Tika Vaani at the beginning of the pilot study developed faith in the program by the end and found the services beneficial.—C33

Behavior improved slowly over time.—C88

Women who did not come for immunization, they now also come for vaccination after listening to the Tika Vaani number.—HW14

### Context

The contextual factors that influenced the fidelity of implementation were classified into two levels:

(i) Implementers: the presence of a motivated fieldwork team that understood the context, had experience in community work, and had the support of supervisors was crucial to implementing the program.
(ii) At the community level, access to mobile phones, level of education, distance from home to meeting places, local weather conditions, and the quality of mobile networks were the main factors that explained the level of community participation and affected the level of fidelity.

We faced difficulties due to the lack of availability of mobile phones for women in the villages.—C33

In the villages, we sometimes met people who had taken alcohol. It was very challenging to make them understand.—C66

It was difficult to give information about TikaVaani to the less educated women of the villages.—C77

Problem is only of mobile network otherwise no problem was faced. We get information free of cost, no money deducted on incoming calls.”—Community#12

Today we cannot meet with men here. Because only a few days ago, the rain had occurred due to which all the men were engaged in the work of their fields.—R-FDBK

### Control group

The main study presents an evaluation of this dimension. According to the analysis of the records, only people who belonged to the intervention group attended the community meetings. The people who communicated with or received calls from the IVR platform were from the intervention group, except for one (1/166) control group member.

## Discussion

We used an established conceptual framework and a mixed methods design to study implementation fidelity as part of a randomized pilot trial. Fidelity assessment shed light on the trial interventions, demonstrating that the Tika Vaani intervention was implemented with high fidelity, supporting the conclusion that the study results faithfully reflected the underlying program theory and demonstrating acceptability and interest to participants. At a methodological level, our study corroborates the importance of assessing the fidelity of pilot projects before assessing whether activities are viable and optimize the intervention before promoting its large-scale implementation [17].

### Factors affecting and facilitating implementation fidelity in Tika Vaani intervention

The modified conceptual framework for implementation fidelity adopted in this study proved useful for assessing fidelity and identifying factors that affect it. The integration of this framework in the study protocol enabled a structured assessment of the key dimensions of adherence (content, coverage, frequency, and duration) and comparison of targeted versus achieved levels of implementation. Moreover, it facilitated structured data collection and analysis of a rich range of interrelated moderating factors that together influenced fidelity. Among the moderating factors, recruitment, participant responsiveness, and context were especially relevant to understanding the level of coverage. The comprehensiveness of the policy description, strategies to facilitate implementation, and quality of delivery had more influence on the level of fidelity achieved. Consistent with other studies, quantitative and qualitative findings converged to reveal how some factors can affect implementation fidelity at *different levels*:

- Implementers of the intervention: The characteristics of implementers, especially their levels of knowledge, experience, motivation, and perception of the intervention, facilitated implementation and helped maintain the high level of fidelity [11, 32, 47, 48]. Additionally, the implementers in the communities gained the people’s trust over time, thereby increasing their confidence in doing the job.
  Beneficiaries: Availability of resources, geographic location, access to mobile phones, and the women’s level of education were barriers to implementing activities and influenced participation in meetings and mHealth components. In this study, gender influenced the effectiveness of both components of the program.
- Program designers: A robust conceptual framework was useful to develop training modules, create manuals, hold periodic follow-up meetings, facilitate implementation, and maintain a high level of fidelity. Therefore, although complex interventions tend to have a lower level of fidelity [49], fidelity can be maintained when programs are well-founded and their theory is understood by implementers [50].

The study of implementation fidelity allowed us to identify these and other programmatic and contextual factors that could affect the results of large-scale interventions [11, 33, 47, 48], and to take steps to strengthen the intervention design.

### Considerations to improve the future intervention and optimize scale-up

Approaches focused on community participation and mHealth are known to improve childhood vaccination coverage [51–54]. However, more evidence is needed regarding mHealth in LMICs facing immunization barriers [55, 56]. This intervention showed that mHealth projects in conditions of limited resources might be useful in improving health knowledge and childhood vaccination [28]. However, in the context of this study, it was not possible to reach the entire community using mobile phones alone. Combining community mobilization with access via mobile phone was essential to increase participation and reach the entire target population. Considering the context where the intervention is implemented is important to discover and understand the variations in the effects of the interventions [57].

Developing context-based approaches combined with complementary actions that increase the probability of reaching different community groups and reduce health disparities is fundamental to achieve the Sustainable Development Goals [58, 59]. Fidelity assessment allows anticipating changes and identifying adjustments that can be made before implementing large-scale interventions [60]. Adjustments based on context should be made to improve program effectiveness and maintain a high level of fidelity [32, 33, 61]. The following recommendations can improve the program and ensure intervention fidelity during scale-up:

#### Community mobilization components

- Local leaders are necessary to promote health education and community mobilization [62, 63]. Implementers found that the recruitment process was challenging. Strategies focused on identifying leaders that adopt recruitment strategies that favor community participation in the long term should be stressed to attract participants to join the different activities.

- Fathers play a significant role in improving the health of their children and stimulating childhood vaccination [64]. Parents who participated in our study acknowledged the usefulness of receiving health messages through a mobile phone and liked the small meetings. Scheduling meetings that favor paternal participation without interfering with work activities improve community involvement and increase awareness about the role of women in decision-making about children’s health [65]. In turn, this approach could increase phone access to women through which they can receive information and increase coverage.

- Geographical location influenced the participation of women in meetings. Continuing to organize meetings in different geographical areas to reach more women will be fundamental to maximize the participation of mothers and empowering them in decision-making about childhood vaccination and increasing vaccination coverage [66].
- The role of health workers in promoting childhood vaccination in large-scale programs to improve the health of less-favored populations is widely recognized [67, 68]. The involvement of these professionals is critical to developing contextualized work agendas that favor their recruitment and engagement over time. Other strategies, such as certified training and non-monetary incentives, including free access to mobile phones or refreshments throughout the intervention period, can be adopted to stimulate health workers’ involvement [69].

#### Messages via mobile phone component

- Acceptance of technology to improve behavior is necessary to promote technological use [70, 71]. The use of mobile phones is crucial in large-scale interventions, and this is an opportunity to improve its functionality. For instance, given the active participation of frontline workers and their acceptance of mobile devices, this technology could be useful to provide training, facilitate data collection to plan vaccination schedules, and improve community health worker performance [72–74]. This approach involves training, improving access to telephones, and developing policies that favor the exchange of information through mobile platforms. The involvement and commitment of different stakeholders can enhance the success of this strategy and optimize the activities of health workers as mediators between the health system and community [69, 73].

### Strengths and limitations

Given the complexity of public health interventions, this study reinforces the value of using mixed methods to assess the fidelity of implementation [47, 75, 76]. The triangulation of multiple sources, data validation, and the rigorous methodology used for analyzing data led to a richer understanding of the findings and the generation of reliable recommendations for a large-scale study. This strategy involves organizing time and mobilizing resources, but the importance of implementing evidence-based actions justifies its planning to reduce costs of ineffective and unreliable programs over time [47]. One main limitation using triangulation design is the discrepancies between different types of data. However, the framework used allows us to develop instruments for data collection in a complementary manner; therefore, findings were not contradictory, and minimal differences could be logically reconciled. Data were collected—by a researcher not involved in the project to avoid information bias—immediately after completing the program to avoid recall bias [77]. The analyses were conducted by an expert in the program, who did not participate in the implementation to ensure independence in the analysis and presentation of results.

The implementers who conducted the community interviews have delivered the mobilization component, possibly favoring a relationship with the participants around the intervention and social desirability bias [77]. However, the evaluation was formative, and the presence of implementers allowed examining the experiences of participants and the need for adjustments before starting a large-scale intervention. Fidelity was evaluated in a research context to determine short-term feasibility, but results may not be fully comparable with real-life situations to detect the factors affecting fidelity. However, the research context is an essential phase to assess program fidelity before conducting a large-scale study.

## Conclusion

This study offers an example of how to conduct and report a study of intervention fidelity in the context of a pilot trial. The results showed that the fidelity of the implementation was high and highlighted the role of specific strategies to improve intervention coverage and reach at scale. Our findings reinforce the importance of opening the “black box” to understand the interactions that occur during the implementation phase in a better manner. Further, they demonstrate the importance of conducting fidelity assessment during pilot studies as a key element of the evaluation process to assess the viability of an intervention, refine it, and transfer it with the best possible evidence to other contexts.

## Supplementary information

**Additional file 1.** Good Reporting of A Mixed Methods Study (GRAMMS) checklist.

**Additional file 2.** Data collection tool to evaluate intervention fidelity.

**Additional file 3.** Qualitative Tools.

**Additional file 4.** Guide small group meeting with communities.

**Additional file 5.** Additional information assessing fidelity of the intervention Tika Vaani.

## Data Availability

The datasets used and/or analyzed during the current study are available from the corresponding author on reasonable request.

## Abbreviations

ASHA: Accredited social health activist
AWWs: *Anganwadi* workers
AWW: Helpers (Sahaika)
CRT: Cluster randomized trial
IVR: Interactive voice response mHealth—mobile health
SBCC: Social and behavior change communication
VHND: Village Health Nutrition Day
