# Supplementary material for: Implementation fidelity and acceptability of an intervention to improve vaccination uptake and child health in rural India: a mixed methods evaluation of a pilot cluster randomized controlled trial
Source: Implement Sci Commun. 2020 Oct 8;1:88. doi: 10.1186/s43058-020-00077-7 (PMC7542710; doi:10.1186/s43058-020-00077-7)
Supplement: Supplementary file 1 — Additional file 1. Good Reporting of A Mixed Methods Study (GRAMMS) checklist. [file 43058_2020_77_MOESM1_ESM.docx]

**Additional file 1. Good Reporting of A Mixed Methods Study (GRAMMS) checklist***

| **Guideline** | Section: page |
| --- | --- |
| Describe the justification for using a mixed methods approach to the research question | **Background**  Section:  **Fidelity of the Tika Vaani Intervention**  Lines: 192-198 |
| Describe the design in terms of the purpose, priority and sequence of methods | **Methods**  Section:  **Study Framework to assess Implementation fidelity**  Lines: 241-245 |
| Describe each method in terms of sampling, data collection and analysis | **Methods**  Section:  **Data collection**  **Data analysis**  Lines: 250-341 |
| Describe where integration has occurred, how it has occurred and who has participated in it | **Methods**  Section:  **Data integration**  Lines: 343-347; Fig. 1. |
| Describe any limitation of one method associated with the present of the other method | **Discussion**  Section:  **Strengths and Limitations**  Lines: 707-709 |
| Describe any insights gained from mixing or integrating methods | **Discussion**  Section:  **Strengths and Limitations**  Lines:  700-706 |

*O'Cathain A, Murphy E, Nicholl J. The quality of mixed methods studies in health services research. J Health Serv Res Policy. 2008;13: 92-98.
