# Supplementary material for: Implementation fidelity and acceptability of an intervention to improve vaccination uptake and child health in rural India: a mixed methods evaluation of a pilot cluster randomized controlled trial
Source: Implement Sci Commun. 2020 Oct 8;1:88. doi: 10.1186/s43058-020-00077-7 (PMC7542710; doi:10.1186/s43058-020-00077-7)
Supplement: Supplementary file 2 — Additional file 2. Data collection tool to evaluate intervention fidelity. [file 43058_2020_77_MOESM2_ESM.docx]

| **Content:**  **Task (planned)** | **Task (completed)** | | **Coverage** | **Frequency** | **Duration** | **Comment** |
| --- | --- | --- | --- | --- | --- | --- |
|  | YES (1) | NO (0) | (I) Implemented as planned  (M) Modified  (C) Cancelled | (I) Implemented as planned  (M) Modified  (C) Cancelled | (I) Implemented as planned  (M) Modified  (C) Cancelled | Please note if necessary: (indicate the date)  a. What difficulties did you face in implementing this activity?  b. What strategies did you use to ensure that planned activities were followed? |
| (Please describe each activity component) |  |  |  |  |  |  |
|  |  |  |  |  |  |  |
|  |  |  |  |  |  |  |
|  |  |  |  |  |  |  |
| **ADAPTATION:** Report if any activity was added during the course of implementation and give reasons | | | | | | |
| **OBSERVATION:** Other pertinent information | | | | | | |

**Additional file 2: Data collection tool to evaluate intervention fidelity***

^*^For each planned activity, assess whether it was: (I) Implemented as planned, (M) Modified or (C) Cancelled
