# Supplementary material for: Implementation fidelity and acceptability of an intervention to improve vaccination uptake and child health in rural India: a mixed methods evaluation of a pilot cluster randomized controlled trial
Source: Implement Sci Commun. 2020 Oct 8;1:88. doi: 10.1186/s43058-020-00077-7 (PMC7542710; doi:10.1186/s43058-020-00077-7)
Supplement: Supplementary file 3 — Additional file 3. Qualitative Tools. [file 43058_2020_77_MOESM3_ESM.docx]

**Additional file 3 Qualitative Tools**

**Tika Vaani Team**

**INFORMED CONSENT FORM**

**Information and Purpose**

You are invited to participate in this interview in order to know if the TV intervention was implemented as planned. The researcher is also interested in the factors that moderate the implementation the Tika Vaani intervention and how Tika Vaani might be improved before applying it at a larger scale. The purpose of this meeting is to gain a better understanding about your experiences implementing Tika Vaani, including what you like about Tika Vaani, what you do not like and if you have any recommendations to improve this intervention.

**Your Participation**

Participation consists of a one-on-one interview and mini discussion groups with TV team, lasting approximately one-hour. You may pass on any question that makes you feel uncomfortable. You also have the right to withdraw from this interview at any time. In the event you choose to withdraw all information you provide (including tapes) will be destroyed and omitted from the final paper

**Benefits and Risks**

The benefit of your participation is to contribute information to improve Tika Vaani intervention before applying it at a larger scale. There are no risks associated with participating in the study.

**Confidentiality**

Our discussion will be audio taped to help accurately capture your insights in your own words. The tapes will only be used for the purpose of this study, however, your name will not be recorded on the tape. Your name and identifying information will not be associated with any part of the written report of the research. All of your information and our discussion will be kept confidential. The researcher will not share your individual responses with anyone other than the research supervisor.If you have any questions or concerns about this study, you may contact me at the e-mail address : [mc.perez.osorio@umontreal.ca](mailto:mc.perez.osorio@umontreal.ca)

**Signature of participant**

I have read the information and consent form. My questions or concerns were resolved. By signing below you agree that you have read and understood the above information, and would be interested in participating in this study.

Signature____________________________________________ Date_______________

**Signature of the person who obtains consent**

I explained to the participant the research project and this consent form and I answered the questions he / she asked me.

Signature____________________________________________ Date_______________

**Tika Vaani Team**

**Interview Guide**

Date__________________________

**Introduction**: Thank you for agreeing to be interviewed. This consent form was necessary for us to ensure that you understand the purpose of your involvement and that you agree to the conditions of your participation.

| 1. **Acceptability and satisfaction concerning the TV intervention** |
| --- |

1. When did you join the Tika Vaani Team?
2. What parts of your job do you find most challenging?
3. What do you find most enjoyable?
4. Do you feel satisfied with the resources you had available to you to deliver the intervention (for example, transport, speakers, pamphlets, paint)?

| 1. **Strategies to facilitate implementation** |
| --- |

1. Did you receive training on how to use the TV platform?

- If yes, it was useful for you?
- Please tell me more about this training

1. Did you receive training on how to contact the community?

- If yes, it was useful for you?
- Please tell me more about this training

1. Did you have manuals or guides to plan your work?

- If yes, it was useful for you?
- Please describe how you used these manuals or guides

1. Did you face any obstacles to promote the TV platform?

If yes

- Could you please give me some examples?
- Please tell me about the strategies that you used to overcome them

| 1. **Quality of delivery** |
| --- |

1. Did you receive feedback from a supervisor during the implementation phase?

- How did you feel about it?

1. Do you feel that there is anything else that supervisors could have done to help you in your work to implement TikaVaani?

- Why?

| 1. **Participant responsiveness** |
| --- |

1. What are some of the positive and negative comments you received from participants about the TV platform?
2. In your opinion, was it easier to promote the participation of people to attend demonstrations and meetings in some villages than in others?

- Why?

1. Can you tell me about your experiences in keeping participants’ attention during community demonstrations and meetings?
2. What techniques did you use to keep participants attention during community demonstrations and meetings?

| 1. **Recruitment** |
| --- |

1. Do you feel that the intervention was able to reach the target population?

- Why?

1. Was the recruitment process consistently applied across all villages?
2. In your opinion, which of these activities were **most appreciated** by the community:

- Community meetings
- Teaching the Tika Vaani platform
- Information capsules in Tika Vaani platform
- Others….

1. In your opinion, which of these activities were **least appreciated** by the community:

- Community meetings
- Teaching the Tika Vaani platform
- Information capsules in Tika Vaani platform
- Others….

1. How did people respond to you when you moved about the community? Were people hostile, or welcoming or neutral?
2. Was this reaction similar towards the beginning and the end of the pilot study?

- If there are any differences, please explain why you think there was a difference?

| 1. **Context** |
| --- |

1. In your view, are there any other important factors related to the local context that might have helped or hindered implementation of the Tika Vaani intervention?

(For example, political issues or policies, leadership, economic factors, disease outbreaks?)

1. Are there any other challenges you faced that we haven’t discussed? Or anything else you would like to add?

##### Thank you for your participation. Your comments, suggestions and ideas are highly appreciated and will be very useful to improve Tika Vaani.

| **Perception concerning the TV intervention (Discussion group)** |
| --- |

In order to know if the field workers understand what is the theory that underlies the intervention, a small group discussion will be held to discuss with the field workers the following questions:

1. Could you tell me about the goals of the Tika Vaani intervention?

2. Do you think these goals are achievable?

- Why?

3. Please describe the activities undertaken to achieve these goals

1. In your opinion, which activities and approaches facilitated the implementation of the Tika Vaani intervention?

- Why do you think these strategies helped? Why?

1. In your opinion, what could be done to improve this intervention?

Which activities would you like to add or exclude? (For example, should there be more or less: visits to the community, information capsules, different topics, different target groups, etc.)

**Note:** A participative approach (interview + discussion group) will create consensus on what is required to improve the intervention and initiate the necessary change/modification, clarify the specific theoretical constructs if it is necessary, in addition, to facilitate assessment of implementation fidelity.

**Community workers (ASHA/ AWWs, AWW Helpers**)

**Interview Guide**

**Village name _________________________**

**Professional information**

- Job title
- Number of years in the profession

**Introduction**: Thank you for agreeing to be interviewed. This consent form was necessary for us to ensure that you understand the purpose of your involvement and that you agree to the conditions of your participation.

| **I. General Information** |
| --- |

During this year did you receive training to improve your work skills?

How did you feel about it?

*If the person has received training

-From whom have you received training?

-In which subjects you have received training?

(exemple : vaccination, drinking water, hand washing, childhood nutrition, etc.)

-Was the training useful or helped you to improve your work skills?

-Would you like to attend these types of training again in future?

1. Would you like to receive training to improve your current work skills?

*If yes

- wthat topic do you like receive ?
- how would you like to receive training?

*If No

- why you do not want to receive training?

1. Would you like to receive information free of cost to improve your work skills by mobile phone?

- If no Why?

| **II. Tika Vaani Intervention Acceptance** |
| --- |

Have you heard about the Tika Vaani intervention? from who?

What do you know about Tika Vaani Intervention?

1. Do you listen the information capsules on the Tika Vaani platform ?

- If yes, with whom do you listen ?
- If no, what is the main reason for not listening ?

1. Have you made a call to the Tika Vaani platform?

***If No**

- What is the main reason why you have not called TV?
- Is there something that would help you to begin using the platform?

##### Go to the question 14.

##### *If Yes go to the question 9

| **II. Experiences and difficulties with the Tika Vaani platform** |
| --- |

1. Please tell me in detail about your experiences using the Tika Vaani platform

For example :

- How did you find the use of the platform?
- Did you have any difficulty communicating or following instructions in the platform?
- Did you find the information easy or difficult to understand?

1. Do you trust the information given on TikaVaani? Why?
2. Do you see any difference in the community after TikaVaani intervention?
3. Do you have any recommendations to improve the content?
4. Do you have any recommendations to improve accessibility or ease of use?
5. Which additional topics or information would you like to be included in the Tika Vaani platform?

| **III. Perceptions and satisfaction the Tika Vaani intervention** |
| --- |

1. Is Tika Vaani helping you in your work, for example, by improving your knowledge, skills, or self-confidence?

*If yes

- Could you give any examples?

*If no

- Why?

1. Did you learn anything new from the Tika Vaani intervention?
2. Would you recommend Tika Vaani platform to members of the community?

- Why?

1. Would you recommend the platform to other health workers?

- Why?

1. In your opinion, what can be done to improve this intervention?
2. Do you have any additional comments or suggestions?

##### Thank you for your participation. Your comments and ideas are greatly appreciated and will be very useful to improve Tika Vaani.

**Community**

**Group Discussion -Instructions**

**Study Team Roles**

Moderator: (name)

Role: Guide the discussion in the group. Be neutral, do not take a position.

Observer : (name)

Role: The observer does not intervene in the discussion. He/She is in charge of the audio recording participants’ answers.

**Moderator’s Introduction**

Good afternoon and welcome to our session. Thank you for taking the time to join us to talk

about Tika Vaani Intervention.

**INTRODUCTION**

My name is ------- and He/She is ----------. We are employees of Jagriti Foundation and represent the study team. We would like to know what you like about Tika Vaani, what you do not like, and how Tika Vaani might be improved.

You were invited to join this discussion because Tika Vaani was offered in this village. We are having discussions like this in all the villages that received Tika Vaani.

Please feel free to share your point of view even if it differs from what others have said. There are no wrong answers but rather differing points of view. We learn from all the different points of view, and sometimes criticism can be the most helpful for learning.

We are going to record the discussion and take notes to be sure that we do not miss any information. Rest assured, your answers will be kept fully confidential.

**Village name: Date:**

**Number of participants:**

- **Women:**
- **Men:**

1. To improve the health of children, Tila Vaani give information through phones and also through community meetings. Is getting information through phones and community meetings is a good method or any other method is better?
2. is it important for families to get the information to improve the health of their children?
3. Those who used TikaVaani mobile phone service, have theyr faced any problem?
4. If yes, what was the biggest problem you faced in using Tika Vaani service? (do not provide options)
5. Was demonstration of Tika Vaani number, getting informtion through mobile phones was clear or understandable?
6. we have information through stories, main messages and direct instruments

6a: was imformation provided on Tika Vaani was clear and understandable?

6b: which method of information dissemination was liked by you the most (stories, main message and direct instruments)

6c: Which is good method for learning?

6d: Do you like to get information through mobile or through community meetings or both?

1. we called two times every week on Monday and Saturday at around 1PM in the afternoon and around 8PM in the night to give information on mobile

Did you find the timing of calls and getting two times calls in a week OK/RIGHT?

How to improve this?

1. we called the parents of children whose vaccination was due, to remind them about vaccination. to remind about vaccination we calles 4 days before vaciination, one day before vacccination and in the morning of vaccination?

8a was reminding families on child vaccination through calls helful?

8b.how can we improve it more?

1. Information given in the community meeting was helpful?
2. For the people who have not used TikaVaani number or never participated in community meetings?

10a. would you please tell us why you people did not call on TikaVaani number or never used it?

10b. if you have young children, then can we do something so that you start using TikaVaani?

1. We have see in few cases, mothers would like to listen the stories on TikaVaani but they did not have phone, their husbands had phones. We can we get the support of family members so that mother can listen to this?
2. If in future, you want to get information on child's health or would like to get any information on any subject like how to make ORS or symptoms of Penumonia or when to do vaccination, you can call on TikaVaani number and listen the information. So do you think this service is helpful?
3. Is there any other topic on which you want to listen messages/information?
4. What do you expect from us to make TikaVaani better?
