# Supplementary material for: Implementation fidelity and acceptability of an intervention to improve vaccination uptake and child health in rural India: a mixed methods evaluation of a pilot cluster randomized controlled trial
Source: Implement Sci Commun. 2020 Oct 8;1:88. doi: 10.1186/s43058-020-00077-7 (PMC7542710; doi:10.1186/s43058-020-00077-7)
Supplement: Supplementary file 4 — Additional file 4. Guide small group meeting with communities. [file 43058_2020_77_MOESM4_ESM.docx]

**Additional file 4 Small group meeting format**

## First small group meeting

**Meeting date:** **Meeting starting time:**

**State name:** Uttar Pradesh **District Name: Hardoi**  **Block Name:** Bawan

**Village Name: Village code:**

**ASHA Name: ASHA phone no.**

**AWW Name: AWW phone no.**

**TikaVaani team members name**

|  | **A. Activities in Village** | | | | | | | | | | | | | | |
| --- | --- | --- | --- | --- | --- | --- | --- | --- | --- | --- | --- | --- | --- | --- | --- |
| **1** | **Meeting with community workers** | | | | | **Does task done** | **Remarks**  **Note: Please comment** | | | **Was the task modified** | | | | **If yes then why and what modification was done** | |
| **a.** | ASHA | | | | | Yes – 1  No – 2 |  | | | Yes – 1  No – 2 | | | |  | |
| **b.** | Aanganwadi worker | | | | | Yes – 1  No – 2 |  | | | Yes – 1  No – 2 | | | |  | |
| **c.** | Aanganwadi helper | | | | | Yes – 1  No – 2 |  | | | Yes – 1  No – 2 | | | |  | |
| **d.** | Were newborn children information collected from Anganwadi worker or ASHA? | | | | | Yes – 1  No – 2 |  | | | Yes – 1  No – 2 | | | |  | |
| **2** | **Visit of selected households** | | | | | **Does task done** | **Remarks**  **Note: Please comment** | | | **Was the task modified** | | | | **If yes then why and what modification was done** | |
| **a.** | Was the team member visited target household to invite them to the small group meeting? | | | | | Yes – 1  No – 2 |  | | | Yes – 1  No – 2 | | | |  | |
| **b.** | Were mobile numbers collected from the selected households? | | | | | Yes – 1  No – 2 |  | | | Yes – 1  No – 2 | | | |  | |
| **c.** | Was the meeting place clam and peaceful? | | | | | Yes – 1  No – 2 |  | | | Yes – 1  No – 2 | | | |  | |
| **3** | Did discussion have with the houses to choose a place for a small group meeting? | | | | | Yes – 1  No – 2 |  | | | Yes – 1  No – 2 | | | |  | |
| **4** | Wall painting in the village | | | | | Yes – 1  No – 2 |  | | | Yes – 1  No – 2 | | | |  | |
| **5** | Poster pasting in the village | | | | | Yes – 1  No – 2 |  | | | Yes – 1  No – 2 | | | |  | |
| **B. Small group activities** | | | | | | | | | | | | | | | |
|  | **Activities** | | | | | **Does task done** | **Remarks**  **Note: Please comment** | | | **Was the task modified** | | | | **If yes then why and what modification was done** | |
| **1.** | Was introduction activity done before starting the meeting? | | | | | Yes – 1  No – 2 |  | | | Yes – 1  No – 2 | | | |  | |
| **2.** | Was TikaVaani number demonstrated? | | | | | Yes – 1  No – 2 |  | | | Yes – 1  No – 2 | | | |  | |
| **3.** | Were TikaVaani slips distributed to everyone in the meeting? | | | | | Yes – 1  No – 2 |  | | | Yes – 1  No – 2 | | | |  | |
| 4. Selected households participation in the meeting | | | | | | | | | | | | | | | |
| **1** | Selected households code in the village  **(Use baseline survey code)** | | | HH Code  _______ | | | HH Code  _______ | | HH Code  _______ | HH Code  _______ | | HH Code  _______ | HH Code  _______ | HH Code  _______ | HH Code  _______ |
|  | **Mother** | | | Yes – 1  No – 2 | | | Yes – 1  No – 2 | | Yes – 1  No – 2 | Yes – 1  No – 2 | | Yes – 1  No – 2 | Yes – 1  No – 2 | Yes – 1  No – 2 | Yes – 1  No – 2 |
|  | **Father** | | | Yes – 1  No – 2 | | | Yes – 1  No – 2 | | Yes – 1  No – 2 | Yes – 1  No – 2 | | Yes – 1  No – 2 | Yes – 1  No – 2 | Yes – 1  No – 2 | Yes – 1  No – 2 |
|  | **Grandmother** | | | Yes – 1  No – 2 | | | Yes – 1  No – 2 | | Yes – 1  No – 2 | Yes – 1  No – 2 | | Yes – 1  No – 2 | Yes – 1  No – 2 | Yes – 1  No – 2 | Yes – 1  No – 2 |
|  | **Grandfather** | | | Yes – 1  No – 2 | | | Yes – 1  No – 2 | | Yes – 1  No – 2 | Yes – 1  No – 2 | | Yes – 1  No – 2 | Yes – 1  No – 2 | Yes – 1  No – 2 | Yes – 1  No – 2 |
|  | **Aunty more than 12 years old (Devrani/Jaithani/nanad)** | | | Yes – 1  No – 2 | | | Yes – 1  No – 2 | | Yes – 1  No – 2 | Yes – 1  No – 2 | | Yes – 1  No – 2 | Yes – 1  No – 2 | Yes – 1  No – 2 | Yes – 1  No – 2 |
|  | **Uncle more than 12 years old (Tau/chacha)** | | | Yes – 1  No – 2 | | | Yes – 1  No – 2 | | Yes – 1  No – 2 | Yes – 1  No – 2 | | Yes – 1  No – 2 | Yes – 1  No – 2 | Yes – 1  No – 2 | Yes – 1  No – 2 |
|  | **Other family members** | | | Yes – 1  No – 2 | | | Yes – 1  No – 2 | | Yes – 1  No – 2 | Yes – 1  No – 2 | | Yes – 1  No – 2 | Yes – 1  No – 2 | Yes – 1  No – 2 | Yes – 1  No – 2 |
| **5. Community workers participation** | | | | | | | | **Yes – 1**  **No – 2** | | | **Remarks**  **Note: Please comment** | | | | |
| ASHA | | | | | | | | Yes – 1 No – 2 | | |  | | | | |
| Aanganwadi worker | | | | | | | | Yes – 1 No – 2 | | |  | | | | |
| Aanganwadi helper | | | | | | | | Yes – 1 No – 2 | | |  | | | | |
|  | |  |  | |  | | | | | | | | | | |
| **New activity** | | | | | | | | | | | | | | | |
| **6. Was new activity added ?** | | | | | **Yes-1 No- 2** | | | | | | | | | | |
| **7. If yes, what activity added and why:** | | | | | | | | | | | | | | | |
|  |  |  |  |  |  |  |  |  |  |  |  |  |  |  |  |
| **8. Did you find any difficulties to implement any activity?** | | | | | **Yes-1 No- 2** | | | | | | | | | | |
| **9. If yes, note the difficulties and activity** | | | | | | | | | | | | | | | |
| **10. What strategies have you used to ensure that programmed activities are followed?** | | | | | | | | | | | | | | | |
| **Please comment:** | | | | | | | | | | | | | | | |
|  |  |  |  |  |  |  |  |  |  |  |  |  |  |  |  |
| **11. Note here about other information that you consider important:** | | | | | | | | | | | | | | | |
|  |  |  |  |  |  |  |  |  |  |  |  |  |  |  |  |
|  |  |  |  |  |  |  |  |  |  |  |  |  |  |  |  |
|  |  |  |  |  |  |  |  |  |  |  |  |  |  |  |  |
| **12. Group discussion General points** | | | | | | | | | | | | | | | |
| 1. Ask to the group participants who all listened to stories on Tika Vani? | | | | | | | | | | | | | | | |
| Number of target household who listened to stories by receiving calls _____  Number of target household who listened to stories by dialing TV number ______  **Please comment_______** | | | | | | | | | | | | | | | |
| 1. Ask to all who all dialed TikaVaani number? | | | | | | | | | | | | | | | |
| Number of target households who dialed TikaVaani number_____  Other households who dialed TikaVaani number_____  **Please comment:______** | | | | | | | | | | | | | | | |
| 1. If dialed number then reason for dialing the number? | | | | | | | | | | | | | | | |
| Please write__ | | | | | | | | | | | | | | | |
| 1. If not listen to TikaVaani stories or not dialed TV number then reason | | | | | | | | | | | | | | | |
| Please write__ | | | | | | | | | | | | | | | |
| 1. If someone listen to TV stories by dialing or receiving call then ask which stories? | | | | | | | | | | | | | | | |
| 1. Pneumonia-write number_________ 2. VHND- write number _______ 3. FAQ2 – write number ______   Please comment:___ | | | | | | | | | | | | | | | |
| 1. If they listened to the stories then what did they learn from the stories? | | | | | | | | | | | | | | | |
| Please write___ | | | | | | | | | | | | | | | |
| 1. The information you got over Tika Vani platform, did you hear that information first time, or you were already know about it? | | | | | | | | | | | | | | | |
| Please write___ | | | | | | | | | | | | | | | |
| 1. If they already know the information, then ask did they know complete information or partial information? | | | | | | | | | | | | | | | |
| Please write___ | | | | | | | | | | | | | | | |
| 1. Ask to the participants, do you believe on information given on TikaVaani? | | | | | | | | | | | | | | | |
| Please write___ | | | | | | | | | | | | | | | |
| 1. Ask to the participants, How were the stories? | | | | | | | | | | | | | | | |
| Please write___ | | | | | | | | | | | | | | | |
| 1. If they liked the stories then ask reason? | | | | | | | | | | | | | | | |
| Please write___ | | | | | | | | | | | | | | | |
| 1. If they did not like the stories then reason | | | | | | | | | | | | | | | |
| Please write___ | | | | | | | | | | | | | | | |
| 1. Did they face any problem in listening the stories or using Tika Vani number? And ask what problems? | | | | | | | | | | | | | | | |
| Please write___ | | | | | | | | | | | | | | | |
| 1. If they faced problem then write problem? | | | | | | | | | | | | | | | |
| Please write___ | | | | | | | | | | | | | | | |
|  | | | | | | | | | | | | | | | |
| **13. Pneumonia capsule** | | | | | | | | | | | | | | | |
| 1. How many of you have heard about Pneumonia? | | | | | | | | | | | | | | | |
| Please write___ | | | | | | | | | | | | | | | |
| 1. Ask the participants, If anyone of you had pneumonia in your household or nearby your household if yes then what had happened or how did you know that it was pneumonia? | | | | | | | | | | | | | | | |
| Please write___ | | | | | | | | | | | | | | | |
| ***Please play pneumonia reminder capsule and then start discussion*** | | | | | | | | | | | | | | | |
| 1. ***Ask, have you ever listened to played information on Tika Vani?*** | | | | | | | | | | | | | | | |
| Please write___ | | | | | | | | | | | | | | | |
| 1. What are the symptoms of Pneumonia? | | | | | | | | | | | | | | | |
| Please write___ | | | | | | | | | | | | | | | |
| 1. What are the benefits of knowing Pneumonia symptoms? | | | | | | | | | | | | | | | |
| Please write___ | | | | | | | | | | | | | | | |
| 1. What were the three steps in the story ? | | | | | | | | | | | | | | | |
| Please write___ | | | | | | | | | | | | | | | |
| 1. How does pneumonia affect the body of children? | | | | | | | | | | | | | | | |
| Please write___ | | | | | | | | | | | | | | | |
| 1. How does Pneumonia spread? | | | | | | | | | | | | | | | |
| Please write___ | | | | | | | | | | | | | | | |
| 1. What are the four measures to prevent pneumonia? | | | | | | | | | | | | | | | |
| Please write___ | | | | | | | | | | | | | | | |
| 1. Ask, after listening story on Pneumonia on TV platform what measures they have taken to fight with Pneumonia? If someone took some measures, then ask everyone to clap their hands so that other can get inspiration. | | | | | | | | | | | | | | | |
| Please write___ | | | | | | | | | | | | | | | |
|  | | | | | | | | | | | | | | | |
|  | | | | | | | | | | | | | | | |
| 1. **VHND capsule** | | | | | | | | | | | | | | | |
| 1. How many of you know about VHND and what do you know? | | | | | | | | | | | | | | | |
| Please write____ | | | | | | | | | | | | | | | |
| 1. If you go to VHND, Why do you go and how is your experience of VHND? | | | | | | | | | | | | | | | |
| Please write___ | | | | | | | | | | | | | | | |
| ***Please play VHND reminder capsule then start discussion*** | | | | | | | | | | | | | | | |
| 1. ***Did they listened this capsule or story on Tika Vani ?*** | | | | | | | | | | | | | | | |
| Please write___ | | | | | | | | | | | | | | | |
| 1. What information have you got from the story? | | | | | | | | | | | | | | | |
| Please write___ | | | | | | | | | | | | | | | |
| 1. On which days VHND organized in the villages according to story? On which day VHND organized in your village? | | | | | | | | | | | | | | | |
| Please write___ | | | | | | | | | | | | | | | |
| 1. What facilities are given in the VHND? | | | | | | | | | | | | | | | |
| Please write___ | | | | | | | | | | | | | | | |
| 1. What are the benefits of having VHND in the village? | | | | | | | | | | | | | | | |
| Please write___ | | | | | | | | | | | | | | | |
| 1. Who all present in VHND to provide health facilities to the people? | | | | | | | | | | | | | | | |
| Please write___ | | | | | | | | | | | | | | | |
| 1. Ask, after listening story on VHND on TV platform, has there been any change in going on vaccination day? what change? | | | | | | | | | | | | | | | |
| Please write___ | | | | | | | | | | | | | | | |
|  | | | | | | | | | | | | | | | |
| **Meeting end time:**___________ | | | | | | | | | | | | | | | |
|  |  |  |  |  |  |  |  |  |  |  |  |  |  |  |  |
|  |  |  |  |  |  |  |  |  |  |  |  |  |  |  |  |
|  |  |  |  |  |  |  |  |  |  |  |  |  |  |  |  |

## **Second small group meeting**

**Meeting date:** **Meeting starting time:**

**State name:** Uttar Pradesh **District Name: Hardoi**  **Block Name:** Bawan

Village Name**: Village code:**

**ASHA Name: ASHA phone no.**

**AWW Name: AWW phone no**

**TikaVaani (TV) team member name(s)**

|  | **A. Activities in Village (RESPONDENT(S): TikaVaani Team Member(s))** | | | | | | | | | | | | | | | |
| --- | --- | --- | --- | --- | --- | --- | --- | --- | --- | --- | --- | --- | --- | --- | --- | --- |
| **1** | **Meeting with community frontline workers** | | | | **Task completed?** | **Remarks**  **Note: Please comment** | | | **Task modified?** | | | | | **If yes, why and what modifications were made?** | | |
| **a.** | ASHA | | | | Yes – 1  No – 2 |  | | | Yes – 1  No – 2 | | | | |  | | |
| **b.** | Anganwadi worker | | | | Yes – 1  No – 2 |  | | | Yes – 1  No – 2 | | | | |  | | |
| **c.** | Anganwadi helper (Sahaika) | | | | Yes – 1  No – 2 |  | | | Yes – 1  No – 2 | | | | |  | | |
| **d.** | Was information on newborn children collected from the Anganwadi worker or ASHA? | | | | Yes – 1  No – 2 |  | | | Yes – 1  No – 2 | | | | |  | | |
| **2** | **Visit to selected (target) households** | | | | **Task completed?** | **Remarks**  **Note: Please comment** | | | **Task modified?** | | | | | **If yes, why and what modifications were made?** | | |
| **a.** | Did the TV team member visit all the target households to invite them to the small group meeting? | | | | Yes – 1  No – 2 |  | | | Yes – 1  No – 2 | | | | |  | | |
| **b.** | Were mobile numbers collected from the selected households? | | | | Yes – 1  No – 2 |  | | | Yes – 1  No – 2 | | | | |  | | |
| **c.** | Was the meeting place calm and peaceful? | | | | Yes – 1  No – 2 |  | | | Yes – 1  No – 2 | | | | |  | | |
| **3** | Did TV team members discuss with households to choose a place for a small group meeting? | | | | Yes – 1  No – 2 |  | | | Yes – 1  No – 2 | | | | |  | | |
| **4** | Poster pasting in the village | | | | Yes – 1  No – 2 |  | | | Yes – 1  No – 2 | | | | |  | | |
| **5** | Wall painting in the village | | | | Yes – 1  No – 2 |  | | | Yes – 1  No – 2 | | | | |  | | |
| **B. Small group activities** | | | | | | | | | | | | | | | | |
|  | **Activities** | | | | **Task completed?** | **Remarks**  **Note: Please comment** | | | **Task modified?** | | | | | **If yes, why and what modifications were made?** | | |
| **1.** | Was the introduction activity done before starting the meeting? | | | | Yes – 1  No – 2 |  | | | Yes – 1  No – 2 | | | | |  | | |
| **2.** | Was the TikaVaani number demonstrated? | | | | Yes – 1  No – 2 |  | | | Yes – 1  No – 2 | | | | |  | | |
| **3.** | Were TikaVaani slips with the phone # distributed to everyone in the meeting? | | | | Yes – 1  No – 2 |  | | | Yes – 1  No – 2 | | | | |  | | |
| 4. **Participation of selected (target) households in the meeting** | | | | | | | | | | | | | | | | |
| **1** | Selected households code in the village  **(Use baseline survey code)** | | | | | HH Code  _______ | | HH Code  _______ | | HH Code  _______ | HH Code  _______ | | HH Code  _______ | | HH Code  _______ | HH Code  _______ |
|  | **Mother** | | | | | Yes – 1  No – 2 | | Yes – 1  No – 2 | | Yes – 1  No – 2 | Yes – 1  No – 2 | | Yes – 1  No – 2 | | Yes – 1  No – 2 | Yes – 1  No – 2 |
|  | **Father** | | | | | Yes – 1  No – 2 | | Yes – 1  No – 2 | | Yes – 1  No – 2 | Yes – 1  No – 2 | | Yes – 1  No – 2 | | Yes – 1  No – 2 | Yes – 1  No – 2 |
|  | **Grandmother** | | | | | Yes – 1  No – 2 | | Yes – 1  No – 2 | | Yes – 1  No – 2 | Yes – 1  No – 2 | | Yes – 1  No – 2 | | Yes – 1  No – 2 | Yes – 1  No – 2 |
|  | **Grandfather** | | | | | Yes – 1  No – 2 | | Yes – 1  No – 2 | | Yes – 1  No – 2 | Yes – 1  No – 2 | | Yes – 1  No – 2 | | Yes – 1  No – 2 | Yes – 1  No – 2 |
|  | **Aunty more than 12 years old (Devrani/Jaithani/nanad)** | | | | | Yes – 1  No – 2 | | Yes – 1  No – 2 | | Yes – 1  No – 2 | Yes – 1  No – 2 | | Yes – 1  No – 2 | | Yes – 1  No – 2 | Yes – 1  No – 2 |
|  | **Uncle more than 12 years old (Tau/chacha)** | | | | | Yes – 1  No – 2 | | Yes – 1  No – 2 | | Yes – 1  No – 2 | Yes – 1  No – 2 | | Yes – 1  No – 2 | | Yes – 1  No – 2 | Yes – 1  No – 2 |
|  | **Other family members** | | | | | Yes – 1  No – 2 | | Yes – 1  No – 2 | | Yes – 1  No – 2 | Yes – 1  No – 2 | | Yes – 1  No – 2 | | Yes – 1  No – 2 | Yes – 1  No – 2 |
| **5. Participation of community workers in the meeting** | | | | | | | **Yes – 1**  **No – 2** | | | | | **Remarks**  **Note: Please comment** | | | | |
| ASHA | | | | | | | Yes – 1 No – 2 | | | | |  | | | | |
| Aanganwadi worker | | | | | | | Yes – 1 No – 2 | | | | |  | | | | |
| Aanganwadi helper | | | | | | | Yes – 1 No – 2 | | | | |  | | | | |
|  | |  |  |  | | | | | | | | | | | | |
| **New activities** | | | | | | | | | | | | | | | | |
| **6. Were any new activities added?** | | | | **Yes-1 No- 2** | | | | | | | | | | | | |
| **7. If yes, which activities were added and why?** | | | | | | | | | | | | | | | | |
|  |  |  |  |  |  |  |  |  |  |  |  |  |  |  |  |  |
| **8. Did you have any difficulties to implement any activity?** | | | | **Yes-1 No- 2** | | | | | | | | | | | | |
| **9. If yes, please note the difficulties and activity** | | | | | | | | | | | | | | | | |
| **10. What strategies have you used to ensure that programmed activities are followed?** | | | | | | | | | | | | | | | | |
| **Please comment:** | | | | | | | | | | | | | | | | |
|  |  |  |  |  |  |  |  |  |  |  |  |  |  |  |  |  |
| **11. Note here any other information that you consider important:** | | | | | | | | | | | | | | | | |
|  |  |  |  |  |  |  |  |  |  |  |  |  |  |  |  |  |
|  |  |  |  |  |  |  |  |  |  |  |  |  |  |  |  |  |
|  |  |  |  |  |  |  |  |  |  |  |  |  |  |  |  |  |
| **C. Group discussion General points (RESPONDENT(S): Community Member(s) – responses recorded by TikaVaani Team Members)** | | | | | | | | | | | | | | | | |
| 1. Ask the group participants: “who all listened to capsules on Tika Vaani?” | | | | | | | | | | | | | | | | |
| Number of target households who listened to capsules by receiving calls _____  Number of other households who listened to capsules by receiving TV number ______  **Please comment_______** | | | | | | | | | | | | | | | | |
| 1. Ask to all who all dialed the TikaVaani number | | | | | | | | | | | | | | | | |
| Number of target households who dialed the TikaVaani number_____  Number of other households who dialed the TikaVaani number_____  **Please comment:______** | | | | | | | | | | | | | | | | |
| 1. For those who dialed the number, then ask “what was the reason for dialing the number?” | | | | | | | | | | | | | | | | |
| Eligible mother/father__  General public____ | | | | | | | | | | | | | | | | |
| 1. For those who did not listen to TikaVaani capsules and did not dial the number, then ask “what was the reason for not listening?” | | | | | | | | | | | | | | | | |
| Eligible mother/father__  General public____ | | | | | | | | | | | | | | | | |
| 1. For those who listened to TikaVaani capsules (either by dialing or receiving calls), then ask “which stories?” | | | | | | | | | | | | | | | | |
| Eligible mother/father__  General public____ | | | | | | | | | | | | | | | | |
| 1. For those who listened to TikaVaani capsules (either by dialing or receiving calls), then ask “what did they learn from the stories?” | | | | | | | | | | | | | | | | |
| Eligible mother/father__  General public____ | | | | | | | | | | | | | | | | |
| 1. For those who listened to TikaVaani capsules (either by dialing or receiving calls), then ask “Did you hear the information you got over the TikaVaani platform for the first time, or did you already know about it?” | | | | | | | | | | | | | | | | |
| Eligible mother/father__  General public____ | | | | | | | | | | | | | | | | |
| 1. If they already knew the information in question 7, then ask “did you already know the complete information, or only partial information?” | | | | | | | | | | | | | | | | |
| Eligible mother/father__  General public____ | | | | | | | | | | | | | | | | |
| 1. For those who listened to TikaVaani capsules (either by dialing or receiving calls), then ask “do you trust the information given on TikaVaani?” | | | | | | | | | | | | | | | | |
| Eligible mother/father__  General public____ | | | | | | | | | | | | | | | | |
| 1. For those who listened to TikaVaani capsules (either by dialing or receiving calls), then ask, “How did you like the stories?” | | | | | | | | | | | | | | | | |
| Eligible mother/father__  General public____ | | | | | | | | | | | | | | | | |
| 1. For those who listened to TikaVaani capsules (either by dialing or receiving calls) and responded to 10 saying that they liked the stories, then ask “What did you like about the stories”? | | | | | | | | | | | | | | | | |
| Eligible mother/father__  General public____ | | | | | | | | | | | | | | | | |
| 1. For those who listened to TikaVaani capsules (either by dialing or receiving calls) and responded to 10 saying that they did NOT like the stories, then ask “Why did you not like the stories”? | | | | | | | | | | | | | | | | |
| Eligible mother/father__  General public____ | | | | | | | | | | | | | | | | |
| 1. Did anyone face any problem in listening to the stories or in using the Tika Vaani number? If so, please ask “what problems?” | | | | | | | | | | | | | | | | |
| Eligible mother/father__  General public____ | | | | | | | | | | | | | | | | |
| 1. For those who responded yes to 13: If they faced any problems, then write “what problem?” | | | | | | | | | | | | | | | | |
| Eligible mother/father__  General public____ | | | | | | | | | | | | | | | | |
| **D. Discussion on Vaccination straight capsules** | | | | | | | | | | | | | | | | |
| ***Part 1: Please play vaccination audio capsule first part and then start discussion*** | | | | | | | | | | | | | | | | |
| 1. In the played capsule, it had been told that vaccines are necessary, do you agree with this? Why do vaccines important for children? | | | | | | | | | | | | | | | | |
| Eligible mother/father__  General public____ | | | | | | | | | | | | | | | | |
| 1. In the played capsule, it had been told that vaccines are necessary for the society, do you agree with this. Why do vaccines important for society? | | | | | | | | | | | | | | | | |
| Eligible mother/father__  General public____ | | | | | | | | | | | | | | | | |
| ***Part 2: Please play vaccination audio capsule second part and then start discussion*** | | | | | | | | | | | | | | | | |
| 1. If a child has been given a few vaccines, will it give full protection to the child? If not then why? | | | | | | | | | | | | | | | | |
| Eligible mother/father__  General public____ | | | | | | | | | | | | | | | | |
| 1. In the played capsule, it had been told that vaccination should be given timely, why should vaccines are given timely? | | | | | | | | | | | | | | | | |
| Eligible mother/father__  General public____ | | | | | | | | | | | | | | | | |
| ***Part 3: Please play vaccination audio capsule third part and then start discussion*** | | | | | | | | | | | | | | | | |
| 1. Vaccines might have some side effect, are these side effect dangerous for the child? | | | | | | | | | | | | | | | | |
| Eligible mother/father__  General public____ | | | | | | | | | | | | | | | | |
| 1. If a child has a minor illness, should the child be given vaccine? Who will decide whether the child should be given a vaccine or not? | | | | | | | | | | | | | | | | |
| Eligible mother/father__  General public____ | | | | | | | | | | | | | | | | |
| 1. If a child is malnourished/weak, should the child be vaccinated? | | | | | | | | | | | | | | | | |
| Eligible mother/father__  General public____ | | | | | | | | | | | | | | | | |
| ***Part 4: Please play vaccination audio capsule fourth part and then start discussion*** | | | | | | | | | | | | | | | | |
| 1. What should be done if a child has never been vaccinated or given a few vaccines? | | | | | | | | | | | | | | | | |
| Eligible mother/father__  General public____ | | | | | | | | | | | | | | | | |
| 1. If a child has been given all the vaccines but still, the child is weak and frequent fall ill, then what is the benefit of vaccination? | | | | | | | | | | | | | | | | |
| Eligible mother/father__  General public____ | | | | | | | | | | | | | | | | |
| ***Part 5: Please play vaccination audio capsule fifth part and then start discussion*** | | | | | | | | | | | | | | | | |
| 1. By what age a child does get vaccines and how many times? | | | | | | | | | | | | | | | | |
| Eligible mother/father__  General public____ | | | | | | | | | | | | | | | | |
| 1. Ask the participants the vaccination schedule? | | | | | | | | | | | | | | | | |
| Eligible mother/father__  General public____ | | | | | | | | | | | | | | | | |
| 1. Where can we see about vaccination? If we want to see which vaccines the child has received and which vaccines are due? | | | | | | | | | | | | | | | | |
| Eligible mother/father__  General public____ | | | | | | | | | | | | | | | | |
| 1. Where can we see about vaccination? If we want to see which vaccines the child has received and which vaccines are due? | | | | | | | | | | | | | | | | |
| Eligible mother/father__  General public____ | | | | | | | | | | | | | | | | |
| 1. Please write if you think anything is important to tell | | | | | | | | | | | | | | | | |
| Eligible mother/father__  General public____ | | | | | | | | | | | | | | | | |
| **Eligible mother/father in the meeting------**  **General public in the meeting-------**  **Meeting end time:**___________ | | | | | | | | | | | | | | | | |
|  |  |  |  |  |  |  |  |  |  |  |  |  |  |  |  |  |
|  |  |  |  |  |  |  |  |  |  |  |  |  |  |  |  |  |
|  |  |  |  |  |  |  |  |  |  |  |  |  |  |  |  |  |

## Third small group meeting

**Meeting date:** **Meeting starting time:**

**State name:** Uttar Pradesh **District Name: Hardoi**  **Block Name:** Bawan

Village Name**: Village code:**

**ASHA Name: ASHA phone no.**

**AWW Name: AWW phone no.**

**TikaVaani (TV) team member name(s)**

|  | | **A. Activities in Village (RESPONDENT(S): TikaVaani Team Member(s))** | | | | | | | | | | | | | | | | |
| --- | --- | --- | --- | --- | --- | --- | --- | --- | --- | --- | --- | --- | --- | --- | --- | --- | --- | --- |
| **1** | | **Meeting with community frontline workers** | | | | **Task completed?** | | **Remarks**  **Note: Please comment** | | | | | **Task modified?** | | | | **If yes, why and what modifications were made?** | |
| **a.** | | ASHA | | | | Yes – 1  No – 2 | |  | | | | Yes – 1  No – 2 | | |  | | | |
| **b.** | | Anganwadi worker | | | | Yes – 1  No – 2 | |  | | | | Yes – 1  No – 2 | | |  | | | |
| **c.** | | Anganwadi helper (Sahaika) | | | | Yes – 1  No – 2 | |  | | | | Yes – 1  No – 2 | | |  | | | |
| **2** | | **Visit to selected (target) households** | | | | **Task completed?** | | **Remarks**  **Note: Please comment** | | | | **Task modified?** | | | **If yes, why and what modifications were made?** | | | |
| **a.** | | Did the TV team member visit all the target households to invite them to the small group meeting? | | | | Yes – 1  No – 2 | |  | | | | Yes – 1  No – 2 | | |  | | | |
| **b.** | | Was the meeting place calm and peaceful? | | | | Yes – 1  No – 2 | |  | | | | Yes – 1  No – 2 | | |  | | | |
| **3** | | Did TV team members discuss with households to choose a place for a small group meeting? | | | | Yes – 1  No – 2 | |  | | | | Yes – 1  No – 2 | | |  | | | |
| **B. Small group activities** | | | | | | | | | | | | | | | | | | |
|  | | **Activities** | | | | **Task completed?** | | **Remarks**  **Note: Please comment** | | | | **Task modified?** | | | **If yes, why and what modifications were made?** | | | |
| **1.** | | Was the introduction activity done before starting the meeting? | | | | Yes – 1  No – 2 | |  | | | | Yes – 1  No – 2 | | |  | | | |
| **2.** | | Was the TikaVaani number demonstrated? | | | | Yes – 1  No – 2 | |  | | | | Yes – 1  No – 2 | | |  | | | |
| **3.** | | Were TikaVaani slips with the phone # distributed to everyone in the meeting? | | | | Yes – 1  No – 2 | |  | | | | Yes – 1  No – 2 | | |  | | | |
| 4. **Participation of selected (target) households in the meeting** | | | | | | | | | | | | | | | | | | |
| **1** | | Selected households code in the village  **(Use baseline survey code)** | | | | | | HH Code  _______ | HH Code  _______ | HH Code  _______ | | | | HH Code  _______ | HH Code  _______ | HH Code  _______ | | HH Code  _______ |
|  | | **Mother** | | | | | | Yes – 1  No – 2 | Yes – 1  No – 2 | Yes – 1  No – 2 | | | | Yes – 1  No – 2 | Yes – 1  No – 2 | Yes – 1  No – 2 | | Yes – 1  No – 2 |
|  | | **Father** | | | | | | Yes – 1  No – 2 | Yes – 1  No – 2 | Yes – 1  No – 2 | | | | Yes – 1  No – 2 | Yes – 1  No – 2 | Yes – 1  No – 2 | | Yes – 1  No – 2 |
|  | | **Grandmother** | | | | | | Yes – 1  No – 2 | Yes – 1  No – 2 | Yes – 1  No – 2 | | | | Yes – 1  No – 2 | Yes – 1  No – 2 | Yes – 1  No – 2 | | Yes – 1  No – 2 |
|  | | **Grandfather** | | | | | | Yes – 1  No – 2 | Yes – 1  No – 2 | Yes – 1  No – 2 | | | | Yes – 1  No – 2 | Yes – 1  No – 2 | Yes – 1  No – 2 | | Yes – 1  No – 2 |
|  | | **Aunty more than 12 years old (Devrani/Jaithani/nanad)** | | | | | | Yes – 1  No – 2 | Yes – 1  No – 2 | Yes – 1  No – 2 | | | | Yes – 1  No – 2 | Yes – 1  No – 2 | Yes – 1  No – 2 | | Yes – 1  No – 2 |
|  | | **Uncle more than 12 years old (Tau/chacha)** | | | | | | Yes – 1  No – 2 | Yes – 1  No – 2 | Yes – 1  No – 2 | | | | Yes – 1  No – 2 | Yes – 1  No – 2 | Yes – 1  No – 2 | | Yes – 1  No – 2 |
|  | | **Other family members** | | | | | | Yes – 1  No – 2 | Yes – 1  No – 2 | Yes – 1  No – 2 | | | | Yes – 1  No – 2 | Yes – 1  No – 2 | Yes – 1  No – 2 | | Yes – 1  No – 2 |
| **5. Participation of community workers in the meeting** | | | | | | | **Yes – 1**  **No – 2** | | | | **Remarks**  **Note: Please comment** | | | | | | | |
| ASHA | | | | | | | Yes – 1 No – 2 | | | |  | | | | | | | |
| Aanganwadi worker | | | | | | | Yes – 1 No – 2 | | | |  | | | | | | | |
| Aanganwadi helper | | | | | | | Yes – 1 No – 2 | | | |  | | | | | | | |
|  | | |  |  |  | | | | | | | | | | | | | |
| **New activities** | | | | | | | | | | | | | | | | | | |
| **6. Were any new activities added?** | | | | | **Yes-1 No- 2** | | | | | | | | | | | | | |
| **7. If yes, which activities were added and why?** | | | | | | | | | | | | | | | | | | |
|  |  |  |  |  |  |  |  |  |  |  |  |  |  |  |  |  |  |  |
| **8. Did you have any difficulties to implement any activity?** | | | | | **Yes-1 No- 2** | | | | | | | | | | | | | |
| **9. If yes, please note the difficulties and activity** | | | | | | | | | | | | | | | | | | |
| **10. What strategies have you used to ensure that programmed activities are followed?** | | | | | | | | | | | | | | | | | | |
| **Please comment:** | | | | | | | | | | | | | | | | | | |
|  |  |  |  |  |  |  |  |  |  |  |  |  |  |  |  |  |  |  |
| **C. Group discussion General points (RESPONDENT(S): Community Member(s) – responses recorded by TikaVaani Team Members)** | | | | | | | | | | | | | | | | | | |
| 1. Ask the group participants: “who all listened to capsules on Tika Vaani?” | | | | | | | | | | | | | | | | | | |
| Number of target households who listened to capsules by receiving calls _____  Number of other households who listened to capsules by receiving TV number ______  **Please comment_______** | | | | | | | | | | | | | | | | | | |
| 1. Ask to all who all dialed the TikaVaani number | | | | | | | | | | | | | | | | | | |
| Number of target households who dialed the TikaVaani number_____  Number of other households who dialed the TikaVaani number_____  **Please comment:______** | | | | | | | | | | | | | | | | | | |
| 1. For those who dialed the number, then ask “what was the reason for dialing the number?” | | | | | | | | | | | | | | | | | | |
| Eligible mother/father__  General public____ | | | | | | | | | | | | | | | | | | |
| 1. For those who did not listen to TikaVaani capsules and did not dial the number, then ask “what was the reason for not listening?” | | | | | | | | | | | | | | | | | | |
| Eligible mother/father__  General public____ | | | | | | | | | | | | | | | | | | |
| 1. For those who listened to TikaVaani capsules (either by dialing or receiving calls), then ask “which stories?” | | | | | | | | | | | | | | | | | | |
| Eligible mother/father__  General public____ | | | | | | | | | | | | | | | | | | |
| 1. For those who listened to TikaVaani capsules (either by dialing or receiving calls), then ask “what did they learn from the stories?” | | | | | | | | | | | | | | | | | | |
| Eligible mother/father__  General public____ | | | | | | | | | | | | | | | | | | |
| 1. For those who listened to TikaVaani capsules (either by dialing or receiving calls), then ask “Did you hear the information you got over the TikaVaani platform for the first time, or did you already know about it?” | | | | | | | | | | | | | | | | | | |
| Eligible mother/father__  General public____ | | | | | | | | | | | | | | | | | | |
| 1. If they already knew the information in question 7, then ask “did you already know the complete information, or only partial information?” | | | | | | | | | | | | | | | | | | |
| Eligible mother/father__  General public____ | | | | | | | | | | | | | | | | | | |
| 1. Do you believe on the information which is given over TikaVaani? | | | | | | | | | | | | | | | | | | |
| Eligible mother/father__  General public____ | | | | | | | | | | | | | | | | | | |
| 1. Ask to the participants, How were the stories? | | | | | | | | | | | | | | | | | | |
| Eligible mother/father__  General public____ | | | | | | | | | | | | | | | | | | |
| 1. If they liked the stories then ask reason? | | | | | | | | | | | | | | | | | | |
| Eligible mother/father__  General public____ | | | | | | | | | | | | | | | | | | |
| 1. If they did not like the stories then reason | | | | | | | | | | | | | | | | | | |
| Eligible mother/father__  General public____ | | | | | | | | | | | | | | | | | | |
| 1. Did they face any problem in listening the stories or using Tika Vani number? And ask what problems? | | | | | | | | | | | | | | | | | | |
| Eligible mother/father__  General public____ | | | | | | | | | | | | | | | | | | |
| 1. If they faced problem then write problem? | | | | | | | | | | | | | | | | | | |
| Eligible mother/father__  General public____ | | | | | | | | | | | | | | | | | | |
|  | | | | | | | | | | | | | | | | | | |
| **D. Diarrhea** | | | | | | | | | | | | | | | | | | |
| 1. Ask to the participants "What is diarrhea?" | | | | | | | | | | | | | | | | | | |
| Eligible mother/father__  General public____ | | | | | | | | | | | | | | | | | | |
| 1. Did your children ever have diarrhea? If yes then how did you know that it was diarrhea and what you did to recover from diarrhea? | | | | | | | | | | | | | | | | | | |
| Eligible mother/father__  General public____ | | | | | | | | | | | | | | | | | | |
| ***Diarrhea management: Please play diarrhea management audio capsule and then start discussion*** | | | | | | | | | | | | | | | | | |  |
| 3) What are the symptoms of diarrhea? | | | | | | | | | | | | | | | | | | |
| Eligible mother/father__  General public____ | | | | | | | | | | | | | | | | | | |
| 4) What is the biggest risk of diarrhea? | | | | | | | | | | | | | | | | | | |
| Eligible mother/father__  General public____ | | | | | | | | | | | | | | | | | | |
| 5) What should be given to children during diarrhea to avoid dehydration? | | | | | | | | | | | | | | | | | | |
| Eligible mother/father__  General public____ | | | | | | | | | | | | | | | | | | |
| 6) Why is it important to avoid dehydration? | | | | | | | | | | | | | | | | | | |
| Eligible mother/father__  General public____ | | | | | | | | | | | | | | | | | | |
| 7) What are the symptoms of severe diarrhea? When should a doctor or hospital be visited for help? | | | | | | | | | | | | | | | | | | |
| Eligible mother/father__  General public____ | | | | | | | | | | | | | | | | | | |
| ***Diarrhea prevention: Please play diarrhea prevention audio capsule and then start discussion*** | | | | | | | | | | | | | | | | | | |
| 8) What are the causes of diarrhea? | | | | | | | | | | | | | | | | | | |
| Eligible mother/father__  General public____ | | | | | | | | | | | | | | | | | | |
| 9) Can diarrhea be prevented? | | | | | | | | | | | | | | | | | | |
| Eligible mother/father__  General public____ | | | | | | | | | | | | | | | | | | |
| 10) How can diarrhea be prevented? | | | | | | | | | | | | | | | | | | |
| Eligible mother/father__  General public____ | | | | | | | | | | | | | | | | | | |
| 11) Why is it important to give mothers milk to children to prevent diarrhea? | | | | | | | | | | | | | | | | | | |
| Eligible mother/father__  General public____ | | | | | | | | | | | | | | | | | | |
| 12) Why is it important to wash hands to prevent diarrhea? | | | | | | | | | | | | | | | | | | |
| Eligible mother/father__  General public____ | | | | | | | | | | | | | | | | | | |
| 13) When should we wash our hands? | | | | | | | | | | | | | | | | | | |
| Eligible mother/father__  General public____ | | | | | | | | | | | | | | | | | | |
| 14) What should be kept in mind while disposing of child feces? | | | | | | | | | | | | | | | | | | |
| Eligible mother/father__  General public____ | | | | | | | | | | | | | | | | | | |
| 15) Which vaccine is about to come to protect from diarrhea? | | | | | | | | | | | | | | | | | | |
| Eligible mother/father__  General public____ | | | | | | | | | | | | | | | | | | |
|  | | | | | | | | | | | | | | | | | | |
| **E. Dengue and Chikungunya** | | | | | | | | | | | | | | | | | | |
| 1. What is dengue and chikungunya? | | | | | | | | | | | | | | | | | | |
| Eligible mother/father__  General public____ | | | | | | | | | | | | | | | | | | |
| 1. Has anyone ever suffered from dengue or chikungunya in your home or neighborhood? If yes, how did you know that it was dengue or chikungunya and what measures were taken that time? | | | | | | | | | | | | | | | | | | |
| Eligible mother/father__  General public____ | | | | | | | | | | | | | | | | | | |
| ***Dengue and Chikungunya prevention: Please play dengue and chikungunya prevention audio capsule and then start discussion*** | | | | | | | | | | | | | | | | | | |
| 3) What measures can be taken to protect from mosquitoes bite? | | | | | | | | | | | | | | | | | | |
| Eligible mother/father__  General public____ | | | | | | | | | | | | | | | | | | |
| 4) Why is it important to remove stagnant water to avoid dengue and chikungunya? | | | | | | | | | | | | | | | | | | |
| Eligible mother/father__  General public____ | | | | | | | | | | | | | | | | | | |
| 5) Why is it necessary to scrub the water container with brushes to avoid dengue and chikungunya? Such as utensils, drums, open buckets etc. used for animal use. | | | | | | | | | | | | | | | | | | |
| Eligible mother/father__  General public____ | | | | | | | | | | | | | | | | | | |
| 6) What are the symptoms of dengue and chikungunya? | | | | | | | | | | | | | | | | | | |
| Eligible mother/father__  General public____ | | | | | | | | | | | | | | | | | | |
| 7) How do dengue and chikungunya spread ? | | | | | | | | | | | | | | | | | | |
| Eligible mother/father__  General public____ | | | | | | | | | | | | | | | | | | |
| ***Dengue and Chikungunya management : Please play dengue and chikungunya management audio capsule and then start discussion*** | | | | | | | | | | | | | | | | | | |
| 8) What measures should be taken after suffering from dengue and chikungunya? | | | | | | | | | | | | | | | | | | |
| Eligible mother/father__  General public____ | | | | | | | | | | | | | | | | | | |
| 9) What should be done to control fever during dengue and chikunguny? | | | | | | | | | | | | | | | | | | |
| Eligible mother/father__  General public____ | | | | | | | | | | | | | | | | | | |
| 10) What is the treatment of dengue and chikunguny? | | | | | | | | | | | | | | | | | | |
| Eligible mother/father__  General public____ | | | | | | | | | | | | | | | | | | |
| 11) If dengue and chikungunya become severe, then what can be the danger? | | | | | | | | | | | | | | | | | | |
| Eligible mother/father__  General public____ | | | | | | | | | | | | | | | | | | |
| 12) What are the symptoms of dengue severity? | | | | | | | | | | | | | | | | | | |
| Eligible mother/father__  General public____ | | | | | | | | | | | | | | | | | | |
|  | | | | | | | | | | | | | | | | | | |
|  | | | | | | | | | | | | | | | | | | |
| **Total eligible mothers in the meeting:-______________ Total other female in the meeting:-_____________**  **Total eligible fathers in the meeting:-____________ Total other male in the meeting:-_________________**  **Desk cheker name:-___________________ Desk check date :-__________________**  **Meeting end time:**___________ | | | | | | | | | | | | | | | | | | |
|  |  |  |  |  |  |  |  |  |  |  |  |  |  |  |  |  |  |  |
|  |  |  |  |  |  |  |  |  |  |  |  |  |  |  |  |  |  |  |
|  |  |  |  |  |  |  |  |  |  |  |  |  |  |  |  |  |  |  |
