# Supplementary material for: Implementation fidelity and acceptability of an intervention to improve vaccination uptake and child health in rural India: a mixed methods evaluation of a pilot cluster randomized controlled trial
Source: Implement Sci Commun. 2020 Oct 8;1:88. doi: 10.1186/s43058-020-00077-7 (PMC7542710; doi:10.1186/s43058-020-00077-7)
Supplement: Supplementary file 5 — Additional file 5. Additional information assessing fidelity of the intervention Tika Vaani. [file 43058_2020_77_MOESM5_ESM.docx]

**Additional file 5 Additional information assessing fidelity of the intervention Tika Vaani**

This document provides an overview of other findings illustrating fidelity assessment for each dimension according to the conceptual framework developed by Carroll et al. (2007) and modified by Hasson et al. (2010).

| **Fidelity Components** | **Summary of results by each component** |
| --- | --- |
| **Adherence** | |
| **Content** | **Community mobilization strategy**  Two villages did not receive a large introductory meeting as planned, but the target families were visited directly to inform them about the intervention.  R-FDBK: “*one (1) village there were only 4 households and, in another (1) village, the team tried to organize the meeting, but people didn’t come to the meeting*.”  Not all villages received the three series of small group meeting (fig a). To compare the experiences among those target families that lived near those who lived far and did not attend the meeting, they were visited at their homes:  *FDBK:”*For small group meeting#2: In two villages it was not conducted because in 1 village there was only 1 household and in the second village there were total 5 household in the village but 3 households had a long distance from each other and our team did individual visits to these households. Our team organized the 1st small group meeting with remaining 2 households because they were very near to each other but in the second small group meeting one target mother (the mother attended first small group meeting) was not present at her home during 2nd small group meeting and our team only did individual visit.*  **For small group meeting#3: in one village had only 1 household and our team did individual visit*.”  **mHealth strategy**  “Pushed” edutainment & summary capsules: they were delivered to people who indicated their interest and had a mobile phone to receive the message or to communicate with the platform.  *Vaccination “reminders”:* they were delivered to the target families, according to the age of each child and if the family had the mobile phone to receive the message.  *‟On-demand”*: this activity comes from the initiative of each participant, so the number of calls was not planned. The platform, despite some technical / network difficulties at the beginning of the intervention, worked without problem during the intervention period.  **Fig. a Number of small group meeting held per village (n=13 villages)** |
| **Coverage** | **Community mobilization strategy**  Community: The implementers did not record the target population during the large introductory meeting, so it is difficult to know precisely how many target family members have directly benefited from the introductory meeting.  -The participation of the community in a small group meeting was represented by 250 women (123 mothers, 88 grandmothers and 39 aunts), 18 men (10 fathers, 6 grandparents and 2 uncles) and 16 children. The participation of the target population in the discussion groups varied during the intervention phase. Overall, 38% (70/184) of families participated in a single meeting, 15.2% (28/184) of families in two meetings while 21.7% (40/184) target families were not present at any meeting. 14% (26/184) of families benefit the all educational capsules as planned through small group meeting.  Health workers: Their participation was low during the entire implementation phase:  -small group meeting # 1: 33 meetings were held, and 30 health workers were invited (ASHAs = 10 AWW = 11 AWH = 9), of which only 5 workers attended (17%). For this first phase of the 33 meetings carried out, 5 meetings were attended by at least 1 health worker.  - Small group meeting # 2: 32 small meetings were held, and 43 health workers were invited (ASHAs = 13 AWW = 16, AWH = 14) of which 12 (28%) attended. For this second phase, of the 32 meetings carried out, 9 meetings were attended by at least one health worker.  -Small group meeting # 3: 31 small meetings were held and 36 health workers were invited (ASHAs = 10 AWW = 11, AWH = 15) of which 7 (27%) attended. For this third and final phase, of the 31 meetings held, 5 meetings were attended by at least one health worker  **mHealth strategy**  For Vaccination "reminders" 22% of target families (40/184) did not have a telephone to receive the message. Of the people who had a mobile phone, 80% (122/144) received at least one reminder message and found it useful:  *Community#2 “there is no other method* [reminding families on child vaccination through calls]*, phone is the best method. ASHA should come and take us”*  *Community#12 “this information* [reminding families on child vaccination through calls] *is very helpful for all.”* |
| **Frequency and duration** | **Community mobilization strategy:**  The frequency of each discussion group took place every four weeks, once the period of four informative capsules were delivered to the community. The average duration of the meetings was between 40 to 60 minutes depending on the interactions and the different topics addressed  **mHealth strategy:**  There was no variation according to the schedule. Of the target families that had a portable cell phone, 56.9% (82/144) heard at least one OBD call (outbound dialing) with an average listening time per household of 151 minutes (range 0 min to 2180 min).  37.5% (54/144) received at least one callback with a listening average of 39 minutes (range between 0 to 1333.7 minutes) per call. The capsule that was most heard by families were the pneumonia capsules.  96.6% (28/29) of health workers have heard at least one capsule with a listening time of 80% or longer. |
| **Moderating factors** | |
| **Comprehensiveness of intervention description** | *-The implementers believe in intervention theory and in the different activities proposed:*  *C55: “Every child’s mother should have information about vaccination and on diseases such as dengue, diarrhoea, etcetera …. If all women understand everything and have full knowledge of vaccination, then our target will be achieved.”*  *C33: “When we make people aware about the vaccination programme through the IVR* [interactive voice response] *and meetings, then (specific) people will definitely get their children vaccinated on time leading to development of a healthy society.” During small meetings, we learned that how much health information the participants got from Tika Vaani…“Creating awareness about health in the community through the medium of the IVR* [interactive voice response] *seems quite effective to me. We can do this on a big scale”*  *C11: “By dialling the Tika Vaani number, people are provided with information on diseases. The Tika Vaani number is free. This is very good, people will receive information about children’s health. If children receive full vaccination, then they will not fall ill due to deadly diseases and will remain healthy.”*  C22: “*Because through these approaches* [doing meetings, providing information through home visits and mobile phone]*, we went to every corner and hamlet and informed people about the Tika Vaani programme so everyone received full information about this program.”*  *C44: “If we keep on informing people in the village about vaccination in this way, then people will become aware.”* |
| **Strategies to facilitate implementation** | The training received and the function’s manual were key elements that facilitated the implementation:  *C33 : “How to use Tika Vaani; How to send an item published on Tika Vaani to a friend or anyone in the community via mobile phone; I received training on the Tika Vaani callback system; How to talk to the Tika Vaani moderator*… It was only by doing all these meetings and activities that our intervention was successful: Through meetings, we were introduced to the people; Through the IVR, people got information about vaccination; Through wall painting, everyone was made aware of the vaccination (Tika Vaani) number; ASHA and Anganwadi, so people gained trust in the Tika Vaani team*”*    C77: “*We developed a plan, made a village map and indicated selected households on it, and with the help of the map were able to reach these households”*  *C88: “[I] worked according to the instructions in the manual … Because of these, it was easy to explain to people”*  Regular meetings were held throughout the implementation phase to verify the planning, evaluate what was implemented and share the difficulties and ways to remedy them. In addition, they used personal records to plan the workday  C77: “*We kept a diary and wrote any plan that we made in it”*  The motivation of the implementers also influenced the implementation of the different activities:  *C22: “I very much liked working in the field (village). In the village, I like discussing with the Pradhan (Mayor) and senior residents.”*  *C33: “Creating awareness about health in the community through the medium of the IVR* [interactive voice response] *seems quite effective to me. We can do this on a big scale”*  *C66: “I very much enjoy meeting and talking with the people in the villages”*  *C88: “I like to inform women about the health of children”*  An element that facilitated community participation was to find a quiet place and use strategies to capture the attention of the participants:  *C33: “We tried to organise meetings in a quiet place. If during the meeting some children created a disturbance, then one of the Tika Vaani team members took the children to a different location and did some activities with them”*  *C77: “To keep the attention of participants we used speakers to dissemination information about Tika Vaani and story telling methods”*  *C88: “First of all, we talked to the women about their daily lives. Afterwards, we would do meetings with them. By this, they would listen carefully to us”*  *C11: “When we asked questions, we used a conversational approach, so that people would not feel that we were asking questions”*  *C66: “During meetings, when we saw that any woman was distracted, we immediately approached her and gave her information about Tika Vaani”* |
| **Quality of delivery** | Before beginning the intervention, the Implementers were trained to balance their knowledge of the different issues addressed to the community.  Knowing the user’s point of view is a way of assessing the quality of the intervention itself. The people who interacted with the platform were dialing the number, receiving call from the platform and who attended the group meetings stated that the information on the content of the different capsule and the way to access the platform was clear, understandable and useful. Likewise, the time in which calls were made to give information and give a reminder message to vaccinate the children was adequate and convenient and they considered it as a very useful strategy to attend the vaccination day.  The health workers found that the information offered on the platform was useful, practical for their work and easy to understand and found no difficulty in entering the platform and following the instructions in each capsule. |
| **Participant responsiveness** | The response of the community and of the health workers was evaluated according to the perception, comprehension and acceptability of the intervention activities, as well as the level of participation in the different activities proposed.  In order to improve women's access to the mobile phone we have asked participants what options could be considered to improve access to information through the platform. Involving men more is an important factor to consider  Community #13 *“women should explain their husband then only husbands allow women to listen the information”*  Community #11 *“explain to men through mobile phones or explain them face to face”*  Community #9 *“when older people listen storied then only, they will inform women”*  Community #12 *“explain our husbands that whenever they get calls, give call to mother of the child”*  Community” #15 *“put the phone near to the household members, eligible mothers will listen to it”*  The lack of time / interest was evoked by some to not participate in community meetings  Community#8 “did not feel the need and did not get the time”  Community#15 *“we do not have time, we do not need this, we do not have money. We do embroidery work and save money, so do not get time, also not have mobile”*  Although there is a better understanding of the importance of childhood vaccination, this is an issue that still presents certain prejudices in some people in the community.  *Community 8: “An eligible mother said that if they go to vaccinated their child her husband scold to her mother-in-law”*  *Community 12 [If a child has been given all the vaccines but still, the child is weak and frequent fall ill, then what is the benefit of vaccination?] “No benefits of vaccination go to doctor”*  The Implementers of the intervention agree that increasing the number of visits to communities and including other health issues can improve not only participation but also the intervention itself  *C44: “There should be capsules to provide information on different diseases. Each village should be visited at least once per month”*  *C66: “More visits to the community, information capsules about different diseases”*  Finally, health workers consider the mobile platform a good strategy to reach the community and other health workers and also suggest that other issues can be attached to be presented to the community  *HW8 “Yes people will get information on vaccination through this number and they will come for vaccination”*  *HW9 “Women in the village ask when their child will get vaccines. Now started giving attention on vaccination and cleanliness”*  *HW3 “now vaccination is happening successful people take their children immediately if their child is sick… Now everyone is coming for vaccination”*  *HW1 “all Anganwadi, ANMs will get support”*  *HW7 “ASHA have to go house to house they will get good information”*  In the same way, the workers express interest to receive training on topics such as child vaccination and family planning and propose as strategies for learning the use of mobile phones, classrooms and video projection. Particularly 76% (13/17) of the health workers interviewed are motivated and would like to receive training through the mobile platform, who do not agree to receive it through this form expressed as the main reason for not having a telephone ( n = 2) or because they don't have time (n = 2). |
| **Recruitment** | The field workers visited each target family to invite them to the different meetings, provide them with educational information and to motivate them to communicate with the platform. Both field workers and health workers believe that the participation of the latter can facilitate the recruitment and participation of the community  HW10: *“Whenever a meeting is organized, the village head, ASHA* [Accredited Social Health Activist]*, Anganwadi worker should be present in the meeting so that women could make aware”* |
| **Context** | The lack of telephone was an important factor that influenced the access of information through this medium:  Community#7 *“our son has mobile phone, so we were not able to listen”*  Community#8 “my husband keeps mobile phones, so we do not get information”  *C77: “Women do not have mobile phones. We faced difficulties when we asked them to dial a number”*  Another contextual factor that influenced the reception of educational messages is the fact of extensive working hours or rainy seasons during the intervention period that did not allow, especially parents, to participate  *R-FDBK*:  *“Men's meeting could not be held because the market was started in this village today…”*  “*Meeting with men was not organized because all the men were busy working on their far*m.”  Apparently, the fact that the platform used the same information at the beginning of each capsule could have been a factor that explains why some parents received the call, but did not listen to the story completely  Community 3: *“Because of same introduction in every capsule an eligible mother used to disconnect TikaVaani call because she thought that she was getting same story*.”  Finally, people change the number very quickly when they receive a text message offering them a new, cheaper card. Then the numbers that were initially registered to receive the information over time are invalid numbers to receive a call, a factor that may explain why some parents who have a telephone did not receive the information as planned. |
| **Grupo control** | The control group received the mobile phone components (OBD, Calback, Reminders) of the intervention one month after the implementation period between September and December 2018 ended. |
